# Homeostatic regulation of renewing tissue cell populations via crowding control: stability, robustness and quasi-dedifferentiation

**DOI:** 10.1007/s00285-024-02057-0

**Published:** 2024-03-23

**Authors:** Cristina Parigini, Philip Greulich

**Affiliations:** 1 School of Mathematical Sciences, University of Southampton, Southampton, UK; 2 Institute for Life Sciences, University of Southampton, Southampton, UK; 3 Te Pūnaha Ātea - Space Institute, University of Auckland, Auckland, New Zealand

**Keywords:** Tissue cell population dynamics, Feedback control, Homeostasis, Stem cells, Stability, Robustness, Dedifferentiation, 92B05

## Abstract

To maintain renewing epithelial tissues in a healthy, homeostatic state, cell divisions and differentiation need to be tightly regulated. Mechanisms of homeostatic regulation often rely on crowding feedback control: cells are able to sense the cell density in their environment, via various molecular and mechanosensing pathways, and respond by adjusting division, differentiation, and cell state transitions appropriately. Here, we determine, via a mathematically rigorous framework, which general conditions for the crowding feedback regulation (i) must be minimally met, and (ii) are sufficient, to allow the maintenance of homeostasis in renewing tissues. We show that those conditions naturally allow for a degree of robustness toward disruption of regulation. Furthermore, intrinsic to this feedback regulation is that stem cell identity is established collectively by the cell population, not by individual cells, which implies the possibility of ‘quasi-dedifferentiation’, in which cells committed to differentiation may reacquire stem cell properties upon depletion of the stem cell pool. These findings can guide future experimental campaigns to identify specific crowding feedback mechanisms.

## Introduction

Many adult tissues are *renewing*, that is, terminally differentiated cells are steadily removed and replaced by new cells produced by the division of cycling cells (stem cells and progenitor cells), which then differentiate. In order to maintain those tissues in a healthy, homeostatic state, (stem) cell divisions and differentiation must be tightly balanced. Adult stem cells are the key players in maintaining and renewing such tissues due to their ability to produce cells through cell division and differentiation persistently (National Institute of Health 2016). However, the underlying cell-intrinsic and extrinsic factors that regulate a homeostatic state are complex and not always well understood.

Several experimental studies have identified mechanisms and pathways that regulate homeostasis. For example, cell crowding can trigger delamination and thus loss of cells in Drosophila back (Marinari et al. 2012), and differentiation in cultured human colon, various zebrafish epithelia, and canine kidney cells (Eisenhoffer et al. 2012; Eisenhoffer and Rosenblatt 2013). On the other hand, cell crowding can affect cell proliferation: overcrowding can inhibit proliferation (Puliafito et al. 2012), whereas a reduction in the cell density, obtained, for example, by stretching a tissue (Gudipaty et al. 2017) causes an increase in proliferative activity (both shown in cultured canine kidney cells). Although the mechanisms to mediate this regulation are not always clear, experimental studies on mechanosensing showed that cell overcrowding generates pressure on cells which they can sense through mechanosensing pathways [e.g. the Hippo pathway (Bin et al. 2011; Yu et al. 2012)], allowing them to reduce cell proliferation (McClatchey and Yap 2012; Puliafito et al. 2012; Shraiman 2005; Hufnagel et al. 2007; Nonomura and Hirata 2020) (“contact inhibition”). Another control mechanism of homeostasis is the competition for limited growth signalling factors (Kitadate et al. 2019; Jörg et al. 2019). More specifically, in the mouse germ line, cells in the stem cell niche respond to a growth factor (FGF5) that promotes proliferation over differentiation, which they deplete upon being exposed to it. Therefore, the more cells are in the niche, the less FGF5 is available per cell, and the less proliferation (or more differentiation) occurs.

Despite differing in the involved molecular pathways and many other details, all these regulatory mechanisms are, in essence, sensing the cell density in their environment, e.g. in the stem cell niche, and responding by adjusting their propensities to divide, differentiate, die, or emigrate from the tissue. This class of mechanisms, for which cell fate propensities depend on the cell density, can be classified as *crowding feedback* regulation: the local cell density determines the cells’ proliferation and differentiation, which affects their population dynamics and thus feeds back to adjust the cell density, in a way that keeps it in a steady state.

Previous studies based on mathematical modelling have shed light on quantitative mechanisms for homeostatic control via feedback when there is a one-way progression of differentiation from stem cells towards terminally differentiated cells (possibly via transient progenitors) (Johnston et al. 2007; Stiehl and Marciniak-Czochra 2011; Bocharov et al. 2011; Lander et al. 2009; Alarcon and Marciniak-Czochra 2011; Stiehl and Marciniak-Czochra 2017). However, it has been shown that differentiation is not always a one-way process, but priming or ’licensing’ for differentiation may occur, which allows cells in early stages of differentiation to return to their naive stem cell state (Ritsma et al. 2014; Hara et al. 2014; Nakagawa et al. 2021; Greulich and Simons 2016; Chatzeli and Simons 2020). In this case, conditions for homeostasis may be more complex than previously proposed. In reference Greulich et al. (2021), necessary conditions for the existence of a homeostatic state have been derived, for the most general case of arbitrary cell state transitions, as well as a sufficient condition for a *dynamic* homeostatic state—which, beyond a strict steady state, allows for bounded oscillations (as in the uterus during the menstrual cycle).

Here, we wish to generalise previous findings about homeostatic control via crowding feedback and identify general conditions for successful control, for possibly complex transitions between cell types and states, including branched cell fate trajectories, reversible switching, and loops. We will consider the situation when propensities for division, differentiation, and loss of (stem) cells are responsive to variations of the cell density in the cellular environment (for example the stem cell niche) and derive conditions that must be minimally fulfilled (necessary conditions) and conditions which are sufficient to ensure that homeostasis prevails. To identify and formulate those conditions, we note that homeostasis is a property of the tissue cell population dynamics, which can be mathematically expressed as a dynamical system. Even if a numerically exact formulation of the dynamics may not be possible, one can formulate generic yet mathematically rigorous conditions by referring to the criteria for the existence of stable steady states in the cell population dynamics of renewing tissues. We will derive those conditions by mathematical, analytical means, augmented by a numerical analysis testing the limits of those conditions.

We will also show that homeostatic control by crowding feedback possesses inherent robustness to failures and perturbations of the involved regulatory pathways, which may occur through external influences (e.g. wide-spread biochemical factors) and genetic mutations. Finally, we will assess the response of cells when the pool of stem cells is depleted. Crucially, we find that upon depletion of the stem cell pool, crowding feedback control causes formerly committed progenitor cells to reacquire self-renewal capacity without substantial changes in their internal states. Dedifferentiation has been widely reported under conditions of tissue regeneration (Donati et al. 2017; Jopling et al. 2011) or when stem cells are depleted (Tata et al. 2013; Tetteh et al. 2015, 2016; Murata et al. 2020), which is usually thought to involve a substantial reprogramming of the cell-intrinsic states towards a stem cell type. On the other hand, our analysis suggests the possibility of “quasi”-dedifferentiation, the reversion of a committed cell to a stem cell by a mere quantitative adjustment of the pacing of proliferation and differentiation, without a substantial qualitative change in its expression profiles.

## Modelling of tissue cell dynamics under crowding feedback

We seek to assess the conditions for homeostasis in renewing tissue cell populations, that is, either a steady state of the tissue cell population (strict homeostasis) or long-term, bounded oscillations or fluctuations (dynamic homeostasis). To this end, we will here derive a formal, mathematical representation of the tissue cell dynamics under crowding feedback regulation.

### Tissue cell population dynamics: a general mathematical framework

The tissue cell population dynamics are defined via the rates of change of cell numbers. Cell numbers in a tissue change via *cell division*, increasing the cell number, and via *cell loss*—either by cell death or shedding from the tissue—which decreases the cell number. Here, we define as a "tissue cell population" a closed population of cells: immigration of cells into this population does not occur, by definition, as the source of this immigration would be included in the here defined tissue cell population. Cell division and loss rates may depend on factors like cell-intrinsic (biochemical and mechanical) states and interactions with other cells, e.g. via paracrine signalling. In principle, cell-intrinsic states could be any molecular or mechanical configuration of the cell, which, from now on, we will call *cell states*. However, to define the cell population dynamics, it is sufficient to distinguish only cell states having different propensities that affect the population dynamics (e.g. different propensities to divide or differentiate). Configurations which are not different in those propensities are pooled together here as one cell state. With this definition, we number those states as $i=1,\ldots ,m$ (*m* can be arbitrarily large) and each state *i* is associated with a unique propensity (i) to divide, $\lambda _i$, (ii) to be lost, $\gamma _i$, or (iii) to change into another cell state $j=1,\ldots ,m$, $\omega _{ij}$. Accordingly, the cell population dynamics are defined by those three processes that each cell may be able to perform. Following the lines of reference Greulich et al. (2021), Parigini and Greulich (2020) and denoting as $X_{i,j,k}$ a cell in cell states *i*, *j*, *k*, respectively, we can formalise this as:

1 $$\begin{aligned} \text{ cell } \text{ division: } X_i&\xrightarrow {\lambda _i r_i^{jk}} X_j + X_k \end{aligned}$$

2 $$\begin{aligned} \text{ cell } \text{ state } \text{ transition: } X_i&\xrightarrow {\omega _{ij}} X_j \end{aligned}$$

3 $$\begin{aligned} \text{ cell } \text{ loss: } X_i&\xrightarrow {\gamma _i}\emptyset \,\,\ , \end{aligned}$$

where the symbols above the arrows denote the dynamical rates of the processes, i.e. the average frequency at which such events occur. Notably, since a cell division may produce daughter cells in different cell states, we assigned to each division the probability $r_i^{jk}$ that a division of a cell in state *i* produces daughter cells in states *j* and *k* ($i=j,j=k,k=i$ are possible), such that $\sum _{j,k=1}^m r_i^{jk} = 1$ for all *i*. In the following, we will denote the total number of cells as *n* and the number of cells in state *i* as $n_i$. The corresponding expected values (mean values) are denoted as ${\bar{n}}$ and ${\bar{n}}_i$, respectively.

The rates given in (1) - (3) denote the *expected* number of events happening per time unit. Thus, we can express the total rate of change of the expected number of cells in state *i*, that is, the derivative $\dot{{\bar{n}}}_i = \frac{d{\bar{n}}_i}{dt}$, in terms of the rates of those events. This defines a set of ordinary differential equations. Following the lines of references Greulich et al. (2021), Parigini and Greulich (2020), we can write $\dot{{\bar{n}}}_i$ as,

4 $$\begin{aligned} \dot{{\bar{n}}}_i = \sum _j \left[ \omega _{ji} + \lambda _j \sum _k \left( r_j^{ik} + r_j^{ki}\right) \right] {\bar{n}}_j - \left( \lambda _i + \gamma _i + \sum _j \omega _{ij}\right) {\bar{n}}_i \, , \end{aligned}$$

where for convenience, we did not write the time dependence explicitly, i.e. ${\bar{n}}_i = {\bar{n}}_i(t)$

In general, the rates $\lambda _i,\gamma _i,\omega _{ij}$ and probabilities $r_i^{jk}$ can depend on the cells and their states in the cellular environment of $X_i$, via paracrine or mechanical signalling. Here, we restrict our study to situations where the explicit spatial position does not matter and where only the number of cells and their states in a close cellular environment affect those parameters. Examples of this type of cell fate regulation are cells competing for signalling molecules in a niche (Jörg et al. 2019; Kitadate et al. 2019) or responding to mechanical pressure and stresses (Puliafito et al. 2012; Shraiman 2005). Yet, more generally, this simplification can also serve as (mean-field) approximation for more complex spatial interactions.

As we examine a situation close to a homeostatic state, we assume that the cell density is homogeneous over the range of interaction between cells, which expands over a volume *V*. Hence, the cell density, $\rho$, is proportional to the expected (average) number of cells in that volume, $\rho = \frac{{\bar{n}}}{V}$. Similarly, the density of cells in cell state *i* is $\rho _i = \frac{{\bar{n}}_i}{V}$.

Thus, the parameters can, in general, depend on the cell densities $\rho _j, j=1,\ldots ,m$. Since *V* is constant, we can divide Eq. (4) by *V* to equivalently express this in terms of the cell state densities, $\rho _i = \frac{{\bar{n}}_i}{V}$, compactly as,

5 $$\begin{aligned} \frac{d}{dt}\varvec{\rho }(t) = A(\varvec{\rho }(t)) \, \varvec{\rho }(t) \, , \end{aligned}$$

where $\varvec{\rho }= (\rho _1,\rho _2,\ldots )$ is the vector of cell state densities and $A(\varvec{\rho })$ is the matrix,

6 $$\begin{aligned} A = \begin{pmatrix} \lambda _1 - \sum _{j \ne 1} \kappa _{1j} - \gamma _1 &{} \kappa _{21} &{} \cdots &{} \kappa _{m1} \\ \kappa _{12} &{} \lambda _2 - \sum _{j \ne 2} \kappa _{2j} - \gamma _2 &{} \cdots &{} \kappa _{m2} \\ \vdots &{} \vdots &{} \ddots &{} \vdots \\ \kappa _{1m} &{} \kappa _{2m} &{} \cdots &{} \lambda _m - \sum _{j \ne m} \kappa _{mj} - \gamma _m \end{pmatrix} \, , \end{aligned}$$

in which $\kappa _{ij} = \lambda _i 2 r_i^j + \omega _{ij}$, with $r_i^j = \sum _k (r_i^{jk}+r_i^{kj})/2$, is the total transition rate, that combines all transitions from $X_i$ to $X_j$ by cell divisions and direct state transitions. Again, all parameters may depend on $\varvec{\rho }$, as therefore also does *A*. We can thus generally write the elements of the matrix *A*, $a_{ij}$ with $i,j=1,\ldots ,m$ as,

7 $$\begin{aligned} a_{ij} = \left\{ \begin{array}{cc} \lambda _i - \gamma _i - \sum _{k \ne i} \kappa _{ik} &{} \text{ for } i = j \\ \kappa _{ji} &{} \text{ for } i \ne j \end{array}\right. \,\,\ . \end{aligned}$$

Finally, we make the mild assumption that the propensities to divide and to change state are controlled separately, that is, the total cell state transition propensities $\kappa _{ij}$ and the cell division rate $\lambda _i$ are the relevant parameters subject to crowding control, as in (6), instead of $\omega _{ij}$ and $r_i^{jk}$.

Equation (5) with matrix A as in (6) describes a dynamical system which, for given initial conditions, determines the time evolution of the cell densities, $\rho _i(t)$. Crucially, this description allows for a rigorous mathematical definition of a homeostatic state and the application of tools of dynamical systems analysis to determine the circumstances under which a homeostatic state prevails. In particular, we define a *(strict) homeostatic state* as a stable non-negative steady state of the system, (5), when the expected cell numbers—and thus cell densities, given that *V* is fixed—in each state do not change, mathematically expressed as $\frac{d{\varvec{\rho }}}{dt} = 0$ and $\rho _i \ge 0$ for all states *i* (a non-negative fixed point of the system). A *dynamic* homeostatic state is when cell densities may also oscillate or fluctuate but remain bounded (Greulich et al. 2021), thus possessing a finite long-term average cell population. Based on these definitions, we can now consider explicit models to analyse under which circumstances crowding feedback can maintain homeostatic states.

### Model constraints, conventions, and conditions for a steady state

To define our particular model, it is helpful to introduce some definitions and conventions and to recapitulate previously established conditions for the existence of a non-negative steady state $\varvec{\rho ^*} = (\rho _1^*,\ldots ,\rho _m^*)$ with $\rho _i^* \ge 0$ for all $i = 1,\ldots ,m$, in a system of the form (5) (Greulich et al. 2019, 2021).

We first note that by choosing an appropriate ordering of the cell states (that is, the basis vectors) the matrix *A* has lower triangular block form (Varga 2000),

8 $$\begin{aligned} A = \left( \begin{array}{ccccc} B_{1} &{}\quad 0 &{}\quad 0 &{}\quad 0 &{}\quad \cdots \\ C_{21} &{}\quad B_{2} &{}\quad 0 &{}\quad 0 &{}\quad \cdots \\ C_{31} &{}\quad C_{32} &{}\quad B_{3} &{}\quad 0 &{}\quad \cdots \\ \vdots &{}\quad \vdots &{}\quad \vdots &{}\quad \ddots &{}\quad 0 \\ \cdots &{}\quad \cdots &{}\quad \cdots &{}\quad \cdots &{}\quad B_{h} \end{array} \right) , \end{aligned}$$

where $B_k$, $k=1,\ldots ,h$, $h \ge 1$, are irreducible matrices. For *A* as in (6), all its off-diagonal elements are non-negative, which means that $B_k$ are irreducible *Metzler matrices*, and the Perron-Frobenius theorem holds for them (MacCluer 2000). This feature implies that each matrix $B_k$ possesses a simple, real maximum eigenvalue $\mu _k$, called the *dominant eigenvalue*. Since *A* is a function of cell densities $\varvec{\rho }$, so is $\mu _k = \mu _k(\varvec{\rho })$. We can define a graph *G*(*A*) for matrix *A*, such that the cell states represent its nodes and the cell state transitions the links between nodes. More precisely, each entry of *A*, $a_{ij}$, corresponds to the weight of the link from node *j* to node *i* in G(A) which, for $j \ne i$, is the transition rate $\kappa _{ji}$ (by definition there is no link from *j* to *i* if $a_{ij}=\kappa _{ji}=0$). *A* can thus be interpreted as the transposed adjacency matrix of *G*(*A*). Note that the diagonal elements of *A* do not change the connectivity of *G*(*A*) and are arbitrarily defined as in (7). The graphs of the irreducible matrices $B_k$, $G(B_k)$, then correspond to the *strongly connected components (SCC)* of *G*(*A*) (Greulich et al. 2019) and the SCCs form a hierarchical structure, without any cycles between different SCCs. We also refer to this as the *lineage hierarchy*.

If $G(B_k)$ does not have any incoming links from other SCCs, then we call it an *apex SCC*, which is the case for $B_1$ and any $B_k$ for which $C_{kl} = \varvec{0}$ for $l < k$; otherwise it is called *non-apex SCC*. In reference Greulich et al. (2021), it was established that a necessary condition for the existence of a non-negative (and non-trivial) steady state in a system of form (5) is that for all apex SCCs, $B_k$ must have dominant eigenvalue $\mu _k = 0$, while all non-apex $B_k$ must have $\mu _k < 0$. Furthermore, if the non-apex matrices $B_k$ do not depend on $\varvec{\rho }$, then this necessary condition is also sufficient for the existence of a non-negative steady state (Greulich et al. 2019).

Following the convention from previous works, we call the collection of all cell states in one SCC of *G*(*A*) a *cell type* [see a detailed discussion of this convention in Greulich et al. (2021)]. Consistently, any state transition from one cell type to another is called *differentiation*. From a biological point of view, apex SCC/cell types which are at a steady state, i.e. maintain a homeostatic population, can be interpreted as *stem cells*, as they have self-renewal potential (the population not changing upon the course of continued divisions) and full lineage potential (being at the apex of the lineage hierarchy) (Greulich et al. 2021). We will make the following **Model Assumptions** to define our model:

1. The parameters of an apex (stem) cell type *s* only depend on the total density of cells in type *s*, that is, elements of an apex SCC $G(B_s)$ only depend on $\rho _s = \sum _{i \in G(B_s)}\rho _i$ (the sum is over all states that constitute the apex cell type). We refer to this interaction as *crowding feedback*.
2. The range of variation of parameters $\lambda _i(\rho _s)$, $\gamma _i(\rho _s)$ and $\kappa _{ij}(\rho _s)$ is sufficiently large, so that a state $\rho ^*_s$ with $\mu _s(\rho ^*_s)=0$ exists for any apex SCC $G(B_s)$.
3. All non-apex cell types $B_k$ have sufficiently high differentiation rates, so that $\mu _k < 0$.

Assumption 1 is based on the common biological scenario where stem cells (apex type cells) reside in a separate niche (Scadden 2006; Watt and Hogan 2000), competing for niche factors that promote self-renewal, or they compete directly for space in the niche, only with each other, and respond to crowding pressure through mechanosensing. In Sect. 3.5, we will consider a similar crowding feedback also for some committed cells. Assumptions 2 and 3 are necessary conditions for the existence of a steady state (Greulich et al. 2021) and are thus always required if we want to assess further (e.g. sufficient) conditions for homeostasis. These conditions mean that the crowding response range and the differentiation rate of non-stem cells are sufficiently high (note that Assumption 3 can always be achieved by a sufficiently high differentiation rate, as increasing $\gamma _i > 0$ decreases the diagonal elements of $B_k$).

With those assumptions, a steady state $\varvec{\rho }^*$ of the whole system prevails if for all apex cell types $B_s$ the corresponding dominant eigenvalue is $\mu _s(\rho ^*_s) = 0$. Hence, in order to determine the condition for homeostasis of the system as a whole, it suffices to determine the conditions for stability of the steady state $\varvec{\rho }^*_s = (\rho ^*_i)\vert _{i \in G(B_s)}$, when the assumptions above are fulfilled. Therefore, for convenience, we will initially restrict our analysis to cell states of an apex cell type/SCC only, neglecting the remainder of the system, whose steady state is assured by the assumption that $\mu _k < 0$ for non-apex SCCs. The inclusion of non-apex SCCs is discussed later in this work. As such, we will in the following sections consider only systems made up of cell states of an apex type $B_s$, and thus identify $B_s$ with *A*, and similarly, $\varvec{\rho _s}$ is denoted as $\varvec{\rho }$ (meaning that $\rho _s$ is denoted as $\rho$, for simplicity), to keep the notation simple.

We note that when we consider only cell states of the apex cell type, any differentiation event (transition to another cell type) is—according to this restricted model—a cell loss event and included as an event occurring with rate $\gamma _i$. Since this corresponds to the irreversible transition to a (committed) non-stem cell type, we will thus denote the rates $\gamma _i$ as *differentiation rates*.

Finally, we choose a convenient notation for model parameters and will often generally refer to them as $\alpha _i$, $i=1,\ldots ,2m+m^2$, where $\alpha _i$ stands for any of the parameters, $\lbrace \lambda _i,\gamma _i,\kappa _{ij}\vert i,j=1,\ldots ,m \rbrace$, respectively^1^. Hence, our main goal is to study which conditions the functions $\alpha _i(\rho )$ must meet to maintain homeostasis. In particular, we study how those parameters qualitatively change with the cell density—increase or decrease—that is, how the signs and magnitudes of derivatives $\alpha _i':=\frac{d\alpha _i}{d\rho }$ affect homeostasis.

### An illustrative simple example

To illustrate the conditions for a strict homeostatic state, which we later wish to generalise, we consider a simple textbook example system [see, e.g. reference Stiehl and Marciniak-Czochra (2011)].

**Fig. 1:**
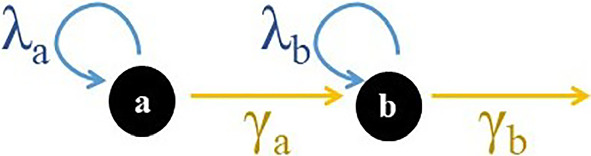
Sketch of the illustrative two-cell state model, (10), (11) under consideration

Let us consider cells with two possible states, *a* and *b*, whereby cells can divide in both states, with rates $\lambda _a$ and $\lambda _b$, respectively, transit from state *a* to state *b*, with rate $\gamma _a$, and be lost from state *b*, with rate $\gamma _b$, according to the following events:

9 $$\begin{aligned} X_a \xrightarrow {\lambda _a} X_a + X_a, \qquad X_a \xrightarrow {\gamma _a} X_b, \qquad X_b \xrightarrow {\lambda _b} X_b + X_b, \qquad X_b \xrightarrow {\gamma _b} \emptyset \,\, , \end{aligned}$$

where $\lambda _a$ and $\gamma _a$ depend on the density of cells in state *a*, $\rho _a$, i.e. $\lambda _a = \lambda _a(\rho _a),\gamma _a=\gamma _a(\rho _a)$ and they are assumed to be monotone functions and thus invertible. The dynamics of this system are written according to (4) as,

10 $$\begin{aligned} \dot{\rho }_a&= \lambda _a(\rho _a) \rho _a - \gamma _a(\rho _a) \rho _a \end{aligned}$$

11 $$\begin{aligned} \dot{\rho }_b&= \gamma _a(\rho _a) \rho _a + \lambda _b \rho _b - \gamma _b \rho _b \,\, , \end{aligned}$$

or alternatively as,

12 $$\begin{aligned} \dot{\varvec{\rho }} = A(\varvec{\rho }) \varvec{\rho }, \text{ with } A(\varvec{\rho }) = \begin{pmatrix} \lambda _a(\rho _a) - \gamma _a(\rho _a) &{} 0 \\ \gamma _a(\rho _a) &{} \lambda _b -\gamma _b \end{pmatrix} \,\,\ , \end{aligned}$$

and $\varvec{\rho } = (\rho _a,\rho _b)$. We can see that *A* has the form $$A=\begin{pmatrix} B_1 &{} 0 \\ C_{21} &{} B_2 \end{pmatrix}$$, where $B_1 = (\lambda _a - \gamma _a)$, $B_2 = (\lambda _b - \gamma _b)$ are $1 \times 1$ (trivially) irreducible matrices. As defined in the previous section, we can associated the matrix *A* with a graph *G*(*A*) where $a_{ij}$ denotes the link weight from node *j* to node *i*. This graph is shown in Fig. 1 (for convenience and clarity, the negative terms on loops are not shown). Here, the states *a* and *b* are trivial one-node SCCs, as no cycles between nodes exist, but in more complex systems these matrices could be larger irreducible matrices corresponding to non-trivial SCCs. Hence, we can identify the states *a* and *b* with SCCs, i.e. *cell types* according to our definition, and a hierarchy prevails: type *a* does not possess incoming links from anywhere else and is therefore an *apex type*, while type *b* possesses an incoming link from type *a* and is a *non-apex type*. Notably, the transition from *a* to *b* is between two cell types, thus it is a *differentiation event* (an irreversible loss of an $X_a$ cell), therefore we had chosen the symbol “$\gamma _a$” instead of “$\omega _{ab}$” for the rate of this event.

For this system, the steady state condition, $\dot{\varvec{\rho }} = 0$, implies $\rho ^*_b = \frac{-\gamma _a(\rho ^*_a)}{\lambda _b - \gamma _b}\rho ^*_a$. A non-trivial and non-negative steady state, $\rho _a^*,\rho _b^* > 0$, can be achieved only if $\lambda _a(\rho ^*_a) - \gamma _a(\rho ^*_a) = 0$ and $\lambda _b - \gamma _b < 0$. We note now that the eigenvalues of the matrix *A* can simply be read off as its diagonal elements, that is, $\mu _a(\rho _a) = \lambda _a(\rho _a) - \gamma _a(\rho _a)$ and $\mu _b = \lambda _b - \gamma _b$. Therefore, the above condition is equivalent to asking for a null dominant eigenvalue of the apex type *a* at the steady state $\rho ^*_a$, $\mu _a(\rho _a^*)=0$ and a negative dominant eigenvalue of the non-apex type *b*, $\mu _b < 0$. This result is consistent with the necessary conditions for a steady state derived in Greulich et al. (2019, 2021), which can only be achieved if Assumptions 2 and 3 from the previous section are met.

The above relations between the model parameters assure the existence of a non-trivial and non-negative steady state, $\varvec{\rho }^*$, but do not specify its stability properties. To determine these, we study the Jacobian matrix at the steady state, *J*, which has the elements,

13 $$\begin{aligned} J_{ij} = \frac{\partial [A(\varvec{\rho }) \varvec{\rho }]_i}{\partial \rho _j} \Bigg \vert _{\varvec{\rho }= \varvec{\rho }^*}, \text{ i.e. } J = \begin{pmatrix} (\lambda _a' - \gamma _a') \rho ^*_a + \gamma _a - \lambda _a &{} 0 \\ \gamma _a'\rho ^*_a + \gamma _a &{} \lambda _b - \gamma _b &{} \end{pmatrix} \,\, , \end{aligned}$$

where here the functions $\lambda _{a},\gamma _a$ are to be taken at $\rho _a^*$, as are the derivatives $\lambda _a',\gamma _a'$.

For $\varvec{\rho }$ to be asymptotically stable it is required that all eigenvalues of *J* are negative. Noting that at the steady state, $\lambda _a - \gamma _a = 0$, the Jacobian matrix *J* has the eigenvalues $(\lambda _a' - \gamma _a')\rho _a^*$ and $\lambda _b - \gamma _b$. Since $\lambda _b - \gamma _b < 0$ is already assured through the steady state condition, the crucial **necessary** and **sufficient** condition for stability is,

14 $$\begin{aligned} \lambda _a' - \gamma _a' < 0\,\, . \end{aligned}$$

Notably, from this we can formulate a simpler, **sufficient condition**,

15 $$\begin{aligned} \lambda _a' \le 0, \,\,\, \gamma _a' \ge 0, \,\, \qquad \lambda _a' \ne 0 \text{ or } \gamma _a' \ne 0 . \end{aligned}$$

The latter condition has the advantage that it only relies on the sign of the feedback, a qualitative condition which is easier to check experimentally.

In the following, we wish to find similar conditions for the stability of steady states, that is, strict homeostasis, in generically more complex systems, with multiple states and non-trivial cell types. As in Eq. (15), we wish to express those conditions in terms of the crowding feedback response, that is, how the parameters $\alpha _i$ (here, $\lambda _a,\gamma _a$) depend on the (stem) cell density $\rho$ (here, $\rho _a$), i.e. the derivatives $\alpha _i'(\rho )$ at the steady state (here, $\lambda _a'(\rho _a),\gamma _a'(\rho _a)$).

## Results

We will now determine necessary and sufficient conditions for the establishment of dynamic and strict homeostasis in renewing cell populations, when subject to crowding feedback, and we will study its stability and robustness. In Sects. 3.1–3.4 we will follow model Assumptions 1–3 from Sect. 2.2 and thus define crowding feedback as dependence of the parameters of stem cells (that is, of an apex cell type as defined above) on the total density of stem cells $\rho := \rho _s = \sum _{i \in G(B_s)}\rho _i$, only. As argued in Sect. 2.2, it suffices to consider stem cells, only, in this case. With this scope, the system constitutes a single cell type (that is a single SCC), thus the matrix *A* is an irreducible Metzler matrix for which a simple dominant eigenvalue $\mu$ and associated eigenvector $\varvec{v}$ exists. In Sect. 3.5 we consider similar assumptions for some non-apex (committed) cell types.

### Sufficient condition for dynamic homeostasis

In Greulich et al. (2021), it was shown that a dynamic homeostatic state, where cell numbers may change over time but stay bounded, is assured if^2^

16 $$\begin{aligned} \mu '(\rho ) < 0 \, \text{ for } \text{ all } \rho > 0 \,\,, \end{aligned}$$

where $\mu$ is the dominant eigenvector of the dynamical matrix of the stem cell type, and $\rho$ the density of stem cells. This sufficient condition requires that $\mu (\rho )$ is a strictly decreasing function of cell density. Also, the range of this function must be sufficiently large so that it has a root, i.e. a value $\rho ^*$ with $\mu (\rho ^*)=0$ must exist for the function $\mu (\rho )$, which is assured by our Assumption 2 from Sect. 2.2.

First, we note that from the Perron-Frobenius theorem follows that each irreducible Metzler matrix, and thus *A*, possesses left and right eigenvectors associated with dominant eigenvalue $\mu$ (MacCluer 2000), respectively indicated as $\varvec{v}$ and $\varvec{w}$, which are strictly positive, that is, all their entries are positive. From this follows that the partial derivative of the dominant eigenvalue $\mu$ by the *i*, *j*-th element of *A*, $a_{ij} = [A]_{ij}$ is always positive:

17 $$\begin{aligned} \frac{\partial \mu }{\partial a_{ij}} = \frac{v_i w_j}{\varvec{v} \varvec{w}} > 0 \,\, , \end{aligned}$$

where the left equality is according to Horn and Johnson (1985) and is generally valid for simple eigenvalues. Here, $\varvec{v}$ is assumed to be in row form, and $\varvec{v} \varvec{w}$ thus corresponds to a scalar product.

According to our assumptions, a non-trivial, non-negative steady state $\varvec{\rho }^*$ exists, and we now translate the sufficient condition for a dynamic homeostatic state, Eq. (16), into conditions on the parameters as a function of the cell density, $\alpha _i(\rho )$. In particular, we can write,

18 $$\begin{aligned} \mu '(\rho ) =&\sum _{ij} \frac{\partial \mu }{\partial a_{ij}} \frac{\partial a_{ij}}{\partial \rho } = \sum _{ij} \frac{v_i w_j}{\varvec{v} \varvec{w}} a'_{ij} = \sum _{i} \frac{v_i w_i}{\varvec{v} \varvec{w}} a'_{ii} + \sum _{i,j \ne i} \frac{v_i w_j}{\varvec{v} \varvec{w}} a'_{ij} \nonumber \\&= \sum _{i} \frac{v_i w_i}{\varvec{v} \varvec{w}} \left( \lambda '_i -\gamma '_i - \sum _{j \ne i}\kappa '_{ij}\right) + \sum _{i,j \ne i}\frac{v_j w_i}{\varvec{v} \varvec{w}}\kappa '_{ij} \,\, , \end{aligned}$$

where we used Eq. (17) and the explicit forms of $a_{ij}$, the elements of the matrix *A*, according to Eq. (7). Provided that all the parameters depend on $\rho$, condition (16) results in:

19 $$\begin{aligned} 0> \mu ' \implies 0> \sum _{i} v_i w_i \left( \lambda '_i - \gamma '_i \right) + w_i \sum _{j\ne i} (v_j - v_i) \kappa '_{ij} \,\,\, \text{ for } \text{ all } \rho > 0 \, , \end{aligned}$$

While we cannot give an explicit general expression for the dominant eigenvectors $\varvec{v}, \varvec{w}$, this condition is sufficiently fulfilled if each term of the sum on the right-hand side of Eq. (19) is negative. More restrictively, we have Eq. (19) sufficiently fulfilled if,

20 $$\begin{aligned} {\left\{ \begin{array}{ll} \lambda '_i \le 0, \, \gamma '_i \ge 0 \text{ for } \text{ all } i \\ \lambda '_i < 0 \text{ or } \gamma '_i> 0 \text{ at } \text{ for } \text{ least } \text{ one } i\\ \kappa '_{ij} = 0 \text{ for } \text{ all } i, j \end{array}\right. } \qquad \text{ for } \rho >0 \,. \end{aligned}$$

This means that, excluding rates that are zero, which are biologically meaningless, if no state transitions within a cell type are subject to crowding feedback ($\kappa '_{ij}=0$), while all (non-zero) cell division rates depend negatively on $\rho$ ($\lambda '_{i}<0$), and differentiation rates depend positively ($\gamma '_{i}>0$), for all attainable levels of $\rho$, then dynamical homeostasis is ensured.

Alternatively, we can rewrite Eq. (19) as

21 $$\begin{aligned} 0> \sum _{i}\dfrac{v_i w_i}{\varvec{v} \varvec{w}} \left( \lambda '_i - \gamma '_i - \sum _{j\ne i} \kappa '_{ij} + \sum _{j\ne i} \dfrac{v_j}{v_i}\kappa '_{ij}\right) \,\,\, \text{ for } \text{ all } \rho > 0 \, , \end{aligned}$$

which, due to $\frac{v_j}{v_i} > 0$, implies another sufficient condition for dynamic homeostasis:

22 $$\begin{aligned} {\left\{ \begin{array}{ll} \lambda '_i \le 0, \, \gamma '_i \ge 0 \text{ for } \text{ all } i \\ \lambda '_i < 0 \text{ or } \gamma '_i > 0 \text{ at } \text{ for } \text{ least } \text{ one } i \\ \kappa '_{ij} \le 0 \text{ with } |\sum _j \kappa '_{ij} |\le \gamma '_i - \lambda '_i \text{ for } \text{ all } i, j \end{array}\right. } \,. \end{aligned}$$

This condition is less strict than Eq. (20), allowing for some non-zero crowding feedback dependency of state transition rates $\kappa _{ij}$, as long as the crowding feedback strength of the total outgoing transition rate of each state does not outweigh the feedback on proliferation and differentiation rate of that state.

### Necessary condition for strict homeostasis

We now consider the circumstances under which a *strict* homeostatic is maintained, that is, when a non-negative steady state of the cell population exists and is asymptotically stable.

From the Perron-Frobenius theorem and the assumptions made in Sect. 2.2 it follows that there exists a non-negative eigenvector $\varvec{\rho }^{*}$ with $A(\rho ^{*}) \varvec{\rho }^{*} = 0$, which can be chosen by normalisation to fulfil $\sum _{i \in S} \rho ^*_i = \rho ^*$. Thus, $\varvec{\rho }^*$ is a fixed point (steady state) of the cell population system (5). Hence, we need to establish what is required for this state to be asymptotically stable.

To start with, we give the Jacobian matrix *J* of the system (5) at the fixed point $\varvec{\rho }^*$, defined by its elements $J_{ij}$ as,

23 $$\begin{aligned}{}[J]_{ij} = \frac{\partial [A(\rho )\varvec{\rho }]_{i}}{\partial \rho _{j}}\Bigg |_{\varvec{\rho }= \varvec{\rho }^*} = a_{ij} (\rho ^*)+ \eta _i \,\, , \end{aligned}$$

in which $a_{ij} = [A]_{ij}$ is the *i*, *j*-th element of matrix *A* and

24 $$\begin{aligned} \eta _i = \sum _{k} a'_{ik} \rho ^{*}_{k} \,. \end{aligned}$$

Here and in the following, we assume the derivatives to be taken at the steady state, i.e. $a'_{ij}:= \frac{da_{ij}}{d\rho }\vert _{\rho =\rho ^*}$. The eigenvalues of the Jacobian matrix *J* at $\rho ^*$ determine the stability of the steady state $\varvec{\rho }^*$: it is asymptotically stable if and only if the real part of all eigenvalues of $J(\rho ^*)$ is negative.

To establish under which conditions the eigenvalues of *J* have all negative real parts, we note that the eigenvalues are the roots of *J*’s characteristic polynomial. The Routh-Hurwitz theorem (Franklin et al. 2014) states that for a polynomial to have only roots with negative real part, all its coefficients must necessarily be positive. Thus, a necessary condition for $\rho ^*$ to be asymptotically stable is that the coefficients of the characteristic polynomial of *J* are all positive.

Let us start by considering a self-renewing cell type with exactly two cell states being at the apex of a lineage hierarchy. This system has a 2 $\times$ 2 dynamical matrix *A* and Jacobian *J*, whereby *A* is irreducible and has dominant eigenvalue $\mu ^A=0$. The characteristic polynomial of a generic 2$\times$2 matrix, *M*, is,

25 $$\begin{aligned} P^M(s) = s^2 + p_1^M s + p_0^M . \end{aligned}$$

with $p_1^M = -\textrm{tr}(M)$ and $p_0^M = \det (M)$. In particular, since *A* has an eigenvalue zero,

26 $$\begin{aligned} p^A_0 = \det (A) = a_{11}a_{22}- a_{12}a_{21} = 0 . \end{aligned}$$

From this follows that the right and left eigenvectors to the matrix *A* associated with the dominant eigenvalue $\mu ^A=0$, $\varvec{w}$ and $\varvec{v}$, are:

27 $$\begin{aligned} \varvec{w} = \begin{pmatrix} -a_{22} \\ a_{21}\end{pmatrix} \text{ and } \varvec{v} = \begin{pmatrix} -a_{22}&a_{12}\end{pmatrix} \,. \end{aligned}$$

For the Jacobian matrix *J*, we get equivalently,

28 $$\begin{aligned} p^J_0&= \det (J) = \dfrac{(a_{21} - a_{22})(- a_{22}\eta _1 + a_{12}\eta _2)}{a_{22}} = \varvec{v} \varvec{\eta }\dfrac{|\varvec{w} |}{a_{22}} \, , \end{aligned}$$

with the $L^1$-norm $|\varvec{w} |= w_1 + w_2 = -a_{22} + a_{21}$^3^. Here we used the form of *J* in Eq. (23) with $\varvec{\eta }= (\eta _1,\eta _2)$ from (24), as well as the relations (26) and (27), and we factorised the determinant.

From Eq. (18), we can further establish:

29 $$\begin{aligned} \mu '&= \sum _{ij} \dfrac{v_i w_j}{\varvec{v} \varvec{w}} a_{ij}' = \sum _{ij} \frac{|\varvec{w} |}{\rho ^*}\dfrac{v_i \rho ^*_j}{\varvec{v} \varvec{w}} a_{ij}' = \frac{|\varvec{w} |}{\rho ^*}\frac{\varvec{v} \varvec{\eta }}{\varvec{v} \varvec{w}} \end{aligned}$$

30 $$\begin{aligned}&= -\frac{a_{22} p_0^J}{\rho ^* p_1^J a_{22}} \,. \end{aligned}$$

Here, we used that $\varvec{\rho }^*$ is a dominant right eigenvector, and thus $\varvec{\rho }^* = \frac{\rho ^*}{|w |} \varvec{w}$, and furthermore we used the definition of $\eta _i = \sum _j a'_{ij} \rho _j^*$, we substituted Eq. (28), and used that $\varvec{v} \varvec{w} = a^2_{22} + a_{12}a_{21} = - p_1^A a_{22}$. Finally, we get:

31 $$\begin{aligned} p^J_0 = -\mu ' \rho ^* p^A_1 \,. \end{aligned}$$

Notably, we can show that this relation also holds for higher dimensions by explicitly computing the coefficients of characteristic polynomials $p_i^{A,J}$, the eigenvalues and eigenvectors, and then evaluating both sides of the equation. For systems with three states, this can be done analytically, by substituting right hand and left hand sides of Eq. (31) and checking for algebraic equality (see section Sect. A.2). For systems with 4,5, and 6 states we tested relation (31) numerically by generating $N=$1000 random matrices with entries chosen from a uniform distribution^4^. In each case, this relation was fulfilled. Hence we are confident that this relation holds up to 6 states, and it is reasonable to expect this to hold also for larger systems.

Since *A* has a simple dominant eigenvalue $\mu ^A=0$, we can factorise one term from the characteristic polynomial of *A*, $P_A(s)=s Q_A(s)$ knowing that all roots of $Q_A(s)$ are negative. Applying the Routh-Hurwitz necessary condition to $Q_A(s)$, it follows that the coefficients of the polynomial *Q* are all negative, $0 > p_i^Q = p_{i+1}^A$, where $i = 0, 1,\ldots , n-1$. Thus, $p_{1}^A > 0$ and considering that $\rho ^* > 0$ by definition, then for having $p^J_0 > 0$ we must require $\mu ' < 0$. Therefore, a necessary condition for a stable, strict homeostatic state is

32 $$\begin{aligned} 0> \mu ' \implies 0 > \sum _{i} v_i w_i \left( \lambda '_i - \gamma '_i \right) + w_i\sum _{j\ne i} (v_j - v_i) \kappa '_{ij}\Bigg \vert _{\rho =\rho ^*} , \end{aligned}$$

where on the right-hand side, we used Eq. (19). This condition is bound to the validity of Eq. (31), that is, we can show it analytically for up to three states and numerically up to 6 states. Nonetheless, we also expect this to be true for larger systems.

One way to satisfy this necessary condition is if at $\rho =\rho ^*$

33 $$\begin{aligned} {\left\{ \begin{array}{ll} \lambda '_i \le 0, \, \gamma '_i \ge 0 \text{ for } \text{ all } i \\ \lambda '_i < 0 \text{ or } \gamma '_i > 0 \text{ at } \text{ for } \text{ least } \text{ one } i \\ \kappa '_{ij} = 0 \end{array}\right. } . \end{aligned}$$

Notably, the conditions (32) and (33) only differ from the sufficient conditions for dynamic homeostasis, Eqs. (19) and (20), by needing to be fulfilled *only at* the steady-state cell density $\rho ^*$, whereas to ensure dynamic homeostasis, those should be valid for a sufficiently large range of $\rho$.

### Sufficient condition for strict homeostasis

Now we assess under which circumstances a strict homeostatic state is assured to prevail.

First of all, the assumptions from Sect. 2.2 and the necessary conditions from above need to be fulfilled. In particular, the parameter functions $\alpha _i(\rho )$ must have a sufficient range so that $\mu (\rho )$ has a root, $\rho ^*$, with $\mu (\rho ^*)=0$, from which the existence of a steady state follows. The question now is whether we can find sufficient conditions assuring that the fixed point $\varvec{\rho }^{*}$ with $\sum _i \rho ^*_i = \rho ^*$ is asymptotically stable.

To this end, let us define a matrix function $B(\varvec{x})$, $\varvec{x} = (x_1,\ldots ,x_m)$ with $b_{ij}(\varvec{x})=[B]_{ij}(\varvec{x}) = a^*_{ij} + x_i$. Hence, $B(\varvec{x})$ interpolates between $B(\varvec{x} = 0) = A(\rho ^*)$ and $B(\varvec{x} = \varvec{\eta }) = J$, where *J*, the Jacobian matrix, and $\varvec{\eta }= (\eta _1,\eta _2,\ldots ,\eta _m)$ are defined according to (23) and (24), respectively. We consider now the dominant eigenvalue as function of the entries of *B*, $\mu [B]:= \mu (\lbrace b_{ij}\rbrace \vert _{i,j=1,\ldots ,m})$, where the square brackets are chosen to denote the difference from the function $\mu (\rho )$. For sufficiently small $\eta _i$, we can then express the dominant eigenvalue of the Jacobian matrix *J*, $\mu [J]$, relative to the dominant eigenvalues of $A^*:= A(\rho ^*)$ as,

34 $$\begin{aligned} \mu [J]&= \mu [A^*] + \sum _i \frac{\partial \mu }{\partial x_i}\Bigg \vert _{x_i = 0} \, \eta _i + O(\varvec{\eta }^2) \end{aligned}$$

35 $$\begin{aligned}&= \sum _i \frac{\partial \mu _A}{\partial a_{ij}} \eta _i + O(\eta _i^2) \end{aligned}$$

where we used that $\mu [A^*] = \mu _A(\rho ^*) = 0$, and

36 $$\begin{aligned} \frac{\partial \mu }{\partial x_i}\Bigg \vert _{x_i = 0} = \sum _{ij} \frac{\partial \mu }{\partial b_{ij}}\frac{\partial b_{ij}}{\partial x_i}\Bigg \vert _{x_i = 0} = \sum _{ij} \frac{\partial \mu }{\partial a_{ij}}\Bigg \vert _{B=A^*} = \sum _{ij} \frac{\partial \mu _A}{\partial a_{ij}} \,\, , \end{aligned}$$

since for $\varvec{x}= \varvec{0}$, $b_{ij}=a_{ij}$ for all *i*, *j*. Hence, since according to (17), $\frac{\partial \mu _A}{\partial a_{ij}} > 0$, the condition for asymptotic stability of the steady state $\varvec{\rho }^*$, $\mu [J] < 0$ is sufficiently fulfilled if $\eta _i < 0$ for all *i*, and if $\vert \eta _i \vert$ is sufficiently small, e.g., so that for all *i*, $\vert O(\eta _i^2) \vert < \vert \frac{\partial \mu _A}{\partial a_{ij}} \eta _i \vert$, which is achievable since $O(\eta _i^2)/\eta _i \rightarrow 0$ for $\eta _i \rightarrow 0$. Thus, we get a sufficient condition for asymptotic stability of the steady state $\rho ^{*}$:

37 $$\begin{aligned} 0> \eta _i = \rho ^{*}_{i} (\lambda '_{i} - \gamma '_{i} ) + \sum _{k \ne i} (\kappa '_{ki} \rho ^{*}_{k}-\kappa '_{ik}\rho ^{*}_{i}) > -\epsilon _i \text{ for } \text{ all } i \end{aligned}$$

where $\epsilon _i > 0$ is sufficiently small. As this is an asymptotically stable steady state, it corresponds to a strict homeostatic state. In this case, even if the cell numbers are disturbed to some degree, the cell population is regulated to return to the strict homeostatic state.

Notably, condition (37) is fulfilled if,

38 $$\begin{aligned} {\left\{ \begin{array}{ll} \lambda '_i \le 0, \, \gamma '_i \ge 0 \text{ for } \text{ all } i \\ \lambda '_i< 0 \text{ or } \gamma '_i > 0 \text{ at } \text{ for } \text{ least } \text{ one } i \\ \kappa '_{ij} = 0 \\ \text{ and } \vert \lambda '_i \vert , \vert \gamma '_i \vert , < \epsilon '_i \end{array}\right. } \end{aligned}$$

where $\epsilon _i' = \frac{\epsilon _i}{2\rho ^*_i}$. Furthermore, we may soften the condition on $\kappa _{ij}$ to $\frac{\kappa '_{ij}}{\kappa '_{ji}} < \frac{\rho ^*_j}{\rho ^*_i}$ to allow also some degree of feedback in $\kappa _{ij}$.

Crucially, in addition to the qualitative nature of the feedback, determined by the signs of $\lambda _i',\gamma _i'$, the ‘strength’ of the crowding feedback, i.e. the absolute values of $\lambda _i',\gamma _i'$ play a role here. Whilst according to the results shown in Sect. 2.3 and those in “Appendix A”, asymptotic stability is ensured for arbitrary feedback strength for systems with a single or two cell states, i.e. $\epsilon _i=\infty$, for larger systems crowding feedback must not be ‘too strong’, that is, smaller than $\epsilon _i$ (or $\epsilon _i'$, respectively). Moreover, as shown in “Appendix A”, for systems with three cell states, we can assure that $\epsilon _i=\infty$ if certain further conditions are met (see Eq. (A13)). Otherwise, $\epsilon _i$ can be determined implicitly from the roots of a quadratic form, Eq. (A14), and thus stability may depend on the strength of the feedback. In principle, such bounds can also be found for larger systems, but this becomes unpractical due to the algebraic complexity to achieve this.

Note, that the conditions (38) are similar to the sufficient conditions for dynamic homeostasis, (20), but here these conditions only need to be fulfilled at $\rho =\rho ^*$, and we have the additional constraint on the feedback strength.

### Robustness to perturbations and failures

Now, we wish to assess the *robustness* of the above crowding control mechanism, i.e. what occurs if it is disrupted, for example, by the action of toxins, other environmental cues, or by cell mutations. More precisely, we will study what happens if one or more feedback pathways, here characterised as parameters $\alpha _i$ with $\alpha _i' \ne 0$ fulfilling the conditions for (dynamic or strict) homeostatic control, are failing, that is, they become $\alpha '_i = 0$. We will first address the case of globally disrupting factors, i.e. those affecting all cells, and then the case of single-cell mutations. In the latter case, only a single cell would initially show a dysregulated behaviour, yet, if this confers a proliferative advantage, it can lead to hyperplasia and possibly cancer (Tomasetti et al. 2013; Colom and Jones 2016; Rodilla and Fre 2018).

First, we note that the sufficient condition for strict homeostasis, given by Eq. (38), may possess redundancies if $\lambda '_i < 0$ and $\gamma '_i > 0$ for more than one *i*. Then, if the feedback is removed for one or more of these parameters (changing to $\lambda '_i = 0$ or $\gamma '_i = 0$), the sufficient condition for a strict homeostatic state can remain fulfilled as long as at least one $\lambda '_i$ or $\gamma '_i$ remains non-zero. This possible redundancy confers a degree of robustness, meaning that feedback responses can be removed—setting $\alpha '_i = 0$—without losing homeostatic control. Since the necessary condition, Eq. (32), is even less restrictive, tissue homeostasis may tolerate more severe disruptions that reverse some feedback pathways, e.g. switching from $\lambda '_i < 0$ to $\lambda '_i > 0$, as long as other terms in the sum on the right-hand side of (32) compensate for this changed sign, ensuring that the sum as a whole is negative. In any case, it is important to remind the underlying assumption for which a non-trivial steady state exists. If the variability of the kinetic parameters is not sufficient to assure the condition $\mu (\rho ^* = 0)$, the tissue will lose homeostasis as well.

From the above considerations, we conclude that if crowding control applies to more than one parameter $\alpha _i$, that is, $\alpha '_i \ne 0$ with appropriate sign and magnitude, homeostasis is potentially robust to the disruption of feedback response pathways. This may include a simple variation of the feedback function $\alpha '_i$ but also complete feedback failure, leading to $\alpha '_i = 0$.

**Fig. 2:**
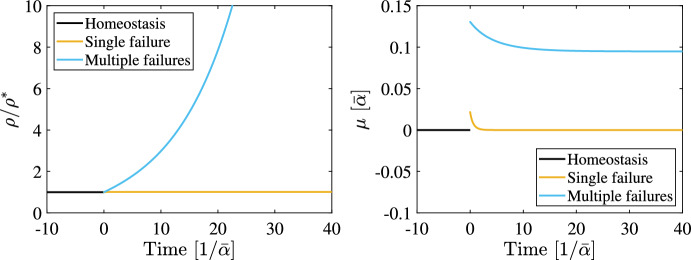
Simulation of robustness. Cell dynamics in terms of cell density, scaled by the steady state in the homeostatic case, as a function of time (left) and the corresponding variation of the dominant eigenvalue $\mu$ (right). Time is scaled by the inverse of $\bar{\alpha } = \min _i{\alpha _i^*}$. The homeostatic model is perturbed at a time equal to zero to include feedback failure by setting $\alpha '_i = 0$ for some *i*. In the case where only one feedback failure occurs (“Single failure”), the system is able to attain a new homeostatic state, characterised by a constant cell density and $\mu =0$. In case more than one feedback fails (“Multiple failures”), the cell dynamics are unstable since a steady state does not exist and $\mu > 0$ for all $\rho$. The simulated model corresponds to model (B15) with parameters given in Table 1 and Table 2

An illustrative example of this situation is shown in Fig. 2. Here, the time evolution of the cell density is shown for a three-state cell fate model, which has been computed by integration of the dynamical system (5) (the details of this model are given in “Appendix B” as Eq. (B15) and illustrated in Fig. 6). Four kinetic parameters are regulated via crowding control satisfying the sufficient condition for strict homeostasis, (38). Then, starting from this homeostatic configuration, feedback disruption is introduced at a time equal to zero. In one case (“Single failure”), a single parameter suffers a complete failure of the type $\alpha _i' = 0$. In this case, the remaining feedback functions compensate for this failure, attaining a new homeostatic state. In contrast, in the second case (“Multiple failures”), failures are applied so that three of the four parameters lose feedback response.^5^ Notably, the only feedback function left satisfies the condition for asymptotic stability, (38). Nevertheless, the variability of this kinetic parameter is not sufficient to assure the existence of a steady state, since, in this case, the function $\mu (\rho )$ does not possess any root. Hence, $\mu > 0$ for all $\rho$, leading to an indefinite growth of the cell population. Additional test cases are presented in “Appendix B.2”.

So far, we modelled the feedback dysregulation as acting on a global scale, thus changing the whole tissue’s dynamics behaviour. However, dysregulation can also act at the single-cell level, for example, when DNA mutations occur. In this case, the impact of the dysregulation is slightly different, as explained in the following.

**Fig. 3:**
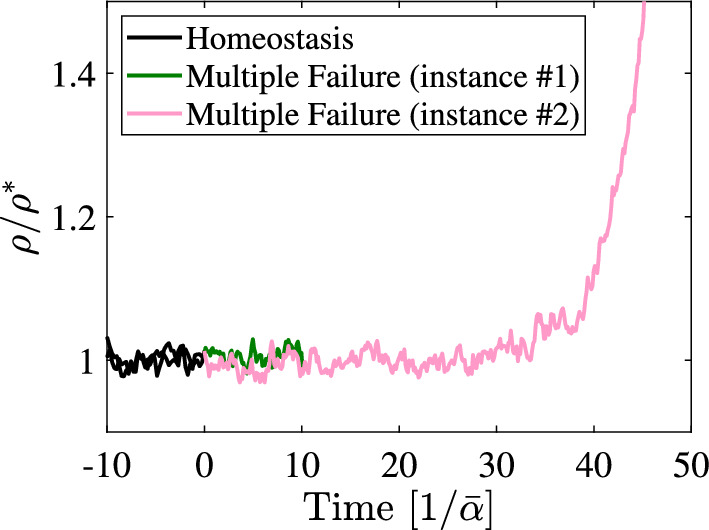
Numerical simulation results of a stochastic version of the model used in Fig. 2 upon disruption of crowding control in a single cell, mimicking a DNA mutation. At a time equal to 0, the initially homeostatic model is disrupted with a single cell presenting multiple failures in the feedback control, as in Fig. 2. Two instances of simulations run with identical parameters are presented. The rescaled cell density $\rho /\rho ^*$ is shown as a function of the time, scaled by the inverse of $\bar{\alpha } = \min _i{\alpha _i^*}$. Whilst the mutated cell and its progeny go extinct in one instance ($\#$1), in the other ($\#$2), mutated cells prevail and hyper-proliferate so that tissue homeostasis is lost. The simulation stops when the clone goes extinct or when instability is detected. Full details of the simulation are provided in “Appendix B.3”

Suppose, upon disruption of crowding control in a single cell, for example, by DNA mutations, a sufficient number of crowding feedback pathways remain so that there is a steady state and the sufficient condition (38) is still fulfilled. In that case, homeostasis is retained, just as when this occurs in a tissue-wide disruption. However, if the homeostatic control of that single cell fails such that the cell becomes hyperproliferative, $\mu > 0$, or declining, $\mu < 0$, the tissue may still remain homeostatic. If $\mu < 0$, the single mutated cell and its progeny will be lost, upon which only a population of crowding-controlled cells remain; that is the population remains homeostatic. If $\mu > 0$ in a single cell, hyper-proliferation is not inevitable either: while the probability for mutated cells to grow in numbers is larger than to decline, there remains a non-zero (and possibly large) probability that the initial single mutated cell is nonetheless lost, out of ’bad luck’, which results in the extinction of the dysregulated mutant.^6^ In that case, the mutant cells go extinct, and the tissue remains homeostatic despite the disruption of homeostatic control in the mutated cells; a stark contrast to disruption on the tissue level. Otherwise, if the mutant clone (randomly) survives, it will continue to hyper-proliferate and eventually dominate the tissue, thus rendering it non-homeostatic. However, the tissue divergence time scale may be much longer than in the case where the same dysregulation occurs in all cells.

To assess the impact of a single-cell mutation on tissue dynamics we choose a stochastic version of the model (B15), as the deterministic model, according to (5), cannot predict random extinction events. To that end, we implemented this situation as a Markov process with the same rates as the tissue cell population dynamics model^7^ (see “Appendix B.3” for more details). In Fig. 3, we show numerical simulation results, depicted in terms of tissue cell density as a function of time. Here, two possible realisations of the same stochastic process are presented. We note that the initially homeostatic tissue exhibits stochastic fluctuations of the cell density, which are around a constant average. At a time equal to zero, a single cell in this tissue switches behaviour, presenting multiple failures which, if applied to all the cells, would determine the growth of the tissue (corresponding to "Multiple Failures" curve in Fig. 2). In one instance of the stochastic simulation, however, the mutated clone goes extinct after some time, leaving the tissue globally unaffected by the mutation. In another instance, the mutated clone prevails, leading to the growth of the tissue cell population. The fact that vastly different macroscopic outcomes can occur with the same parameters and starting conditions demonstrates the impact of stochasticity on large-scale tissue dynamics in the case of a single-cell mutation.

### Quasi-dedifferentiation

Disruption of the tissue may not be restricted to the dysregulation of pathways, but in extreme cases, caused for example by toxins or radiation, the stem cell population as a whole may be depleted. In this context, many studies about tissue regeneration after injury report evidence of cell plasticity (Tetteh et al. 2015, 2016), when committed cells regain the potential of the previously depleted stem cells, generally referred to as *dedifferentiation* (Tata and Rajagopal 2016; Merrell and Stanger 2016; Tata et al. 2013; Puri et al. 2015).

**Fig. 4:**
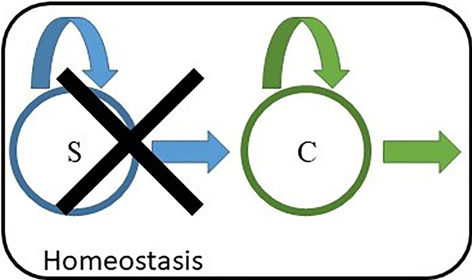
Sketch representative of the quasi-dedifferentiation scenario. A homeostatic system enclosed in the black box comprises two cell types: a stem cell type, *S*, (blue) and a committed cell type, *C*, (green). In the unperturbed homeostatic scenario, *S* is a stem cell, characterised by a growth parameter at the steady state $\mu _s^* = 0$, and *C* is transient, with a growth parameter at the steady state $\mu _c^* < 0$. Both cell types are subject to crowding control, fulfilling both conditions (20), and (38). By removing the stem cell type $X_S$, the committed cell type becomes an apex-type, and thus acquires self-renewing property through crowding control, effectively becoming a stem cell type (see Fig. 5)

In the following, we assess how committed progenitor cells respond to the depletion of the stem cell pool if they are under crowding feedback control. For this purpose, we consider in addition to an apex cell type (S), representing a stem cell type, also a non-apex cell type (C), representing *committed cells*, which resides below type S in the hierarchy, that is, cells of type S can differentiate into type C, as depicted in Fig. 4. We consider the scenario that both cell types are separately subject to crowding control: as in previous sections, S-cells are supposed to reside in a niche and their dynamical parameters $\alpha _i$ only depend on the density of S-cells. On the other hand, dynamics of C-cells may depend on both the densities of S-cells and of C-cells. Note that Assumption (1) in Sect. 2.2 is generalized here to be applicable also to the committed progenitors. The dynamics of density of the C-cells can therefore be expressed as,

39 $$\begin{aligned} \frac{d}{dt}\varvec{\rho }_c = A_c(\rho _s,\rho _c) \varvec{\rho }_c + \varvec{u}(\varvec{\rho }_s) \, , \end{aligned}$$

where $\varvec{\rho }_s = (\rho _{1},\rho _{12},..,\rho _{m_s})$ and $\varvec{\rho }_c = (\rho _{m_s+1},\rho _{m_s+2},..,\rho _{m_s+m_c})$ are the cell densities of S- and C-types, respectively, with ${m_s}$ being the number of states of S. $A_c$ is the part of the matrix *A* that is restricted to states in the C-type (corresponding to $B_2$ in (8)) and $\varvec{u}$ with $u_i = \sum _{j=1}^{m_s}\kappa _{ji} \rho _j$ is the total rate of S-cells differentiating into C-cells.

We further assume that both, the S- and C-type, fulfil the sufficient conditions for dynamic homeostasis, (20), and for stable, strict homeostasis, (38), with respect to cell densities of their own type, and we also assume that both cell types can divide through at least one cell state (i.e. $\lambda _i,\lambda _j > 0$ for at least one $i \in S$ and one $j \in C$), that is, C-cells are committed *progenitor* cells.

Due to crowding control, the density of S-cells, $\varvec{\rho }_s$, is in a stable steady state. Hence, $\varvec{\rho }_s = \varvec{\rho }_s^*$ can be seen as constant and the dynamics of C can be written as,

40 $$\begin{aligned} \frac{d}{dt}\varvec{\rho }_c = A^*_c(\rho _c) \varvec{\rho }_c + \varvec{u}^* \, , \end{aligned}$$

where $$A_c^*(\rho _c) = A_c(\rho _s^*,\rho _c)$$ and $$u^*_i = \sum _{j=1}^{m_s}\kappa _{ji} \rho _j^*$$,

Since $\varvec{\rho }_s^*$ is constant, independently of the state of C-cells, for the Jacobian matrix we only need to consider variations in $\varvec{\rho }_c$, that is, we write the Jacobian matrix as $J=\left[ \frac{\partial A^*(\rho _c) \varvec{\rho }_c}{\partial \rho _j}\right] _{j=m_s + 1,\ldots ,m_s+m_c}$, which has the same form as a cell type at the apex of the hierarchy, since $\varvec{u}^*$ does not depend on the densities $\rho _{m_s+1,\ldots ,m_s+m_c}$. From this follows that if C-cells are regulated by crowding control, fulfilling the conditions (38), then also the population of C-cells is stable around a steady state $\varvec{\rho }_c^*$, albeit with a dominant eigenvalue $\mu _c(\rho _c^*) < 0$.^8^

We now consider the scenario where all stem cells are depleted at some point, as was experimentally done in Tata et al. (2013), Tetteh et al. (2016). This would stop any replenishment of C-cells through differentiation of S-cells, corresponding to setting $$\varvec{u}^*=0$$ in (40). Hence, we end up with the dynamics $$\dot{\varvec{\rho }_c} = A^{**}(\rho _c) \varvec{\rho }_c$$, in which $$ A^{**}(\rho _c) = A(\rho _s=0,\rho _c)$$. We assume here that the function $$\mu _c(\rho )$$ has sufficient range so that $$\mu _c(\rho _c^{**})=0$$ for some $$\rho _c^{**}$$, and that $$A^{**}(\rho _c)$$ is under crowding control fulfilling the sufficient conditions for asymptotic stability of a steady state. Therefore, following our arguments from Sect. 3.3, the population of C-cells will attain a stable steady state, with $$\mu _{A^{**}}(\rho _c^{**}) = 0$$. In other words, those previously committed cells (non-apex type) become stem cells—an apex type with stable steady state population.

Hence, under crowding control, previously committed progenitor cells (committed cells that can divide) will automatically acquire stem cell characteristics if the original stem cells are depleted. Commonly, such a reversion of a committed cell type to a stem cell type would be called ‘dedifferentiation’ or ‘reprogramming’. However, in this case, no genuine reversion of cell states occurs; previously committed cells do not transition back to states associated with the stem cell type. Instead, they respond by crowding feedback and rebalance their dynamical rates so that $\mu$ becomes zero, hence attaining a self-renewing cell type. Crucially, this new stem cell type is fundamentally different to the original stem cells and still most similar to the original committed type. We call this process *quasi-dedifferentiation*. The quasi-dedifferentiation follows the same reversion of proliferative potential as in ‘genuine’ dedifferentiation but without explicit reversion in the cell state trajectories.

**Fig. 5:**
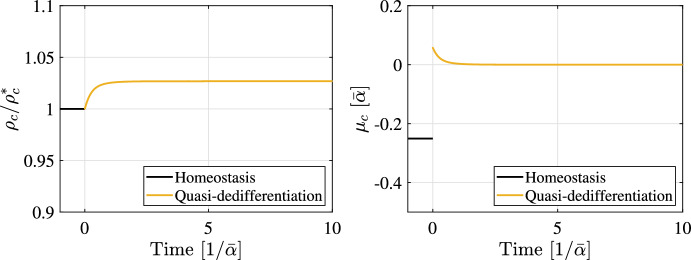
Simulation of quasi-dedifferentiation. Cell dynamics of an initially committed cell type C ($\mu <0$) upon removal of all stem cells. (Left) Cell density scaled by the steady-state density as a function of time. (Right) Corresponding variation of the dominant eigenvalue $\mu _c$. Time is scaled by the inverse of $\bar{\alpha } = \min _i{\alpha _i^*}$. It is assumed that a stem cell type, S, initially resides in the lineage hierarchy above the committed cell type (as in Fig. 4). S cells differentiate into C cells, which is modelled as a constant cell influx of C-cells (S is not explicitly simulated). At a time equal to zero, a sudden depletion of S cells is modelled by stopping the cell influx. After some transitory phase, the cell population stabilises around a new steady state and becomes self-renewing with $\mu _c = 0$. The full description of the dynamical model, which corresponds to model (B15) with parameters given in Table 1, is reported in “Appendix B.4”

**Fig. 6:**
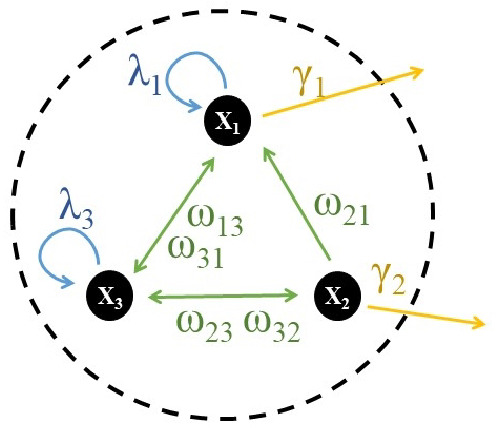
Cell state network representing a cell type composed of three states. The links represent direct transitions, $\omega _{ij}$; symmetric divisions occur with rates $\lambda _i$ and differentiation with rate $\gamma _i$, where subscripts $i,j=1,2,3$ indicate the corresponding cell state, as per model (B15)

The following numerical example illustrates this situation. We focus on the cell dynamics of a single C-type regulated via crowding feedback (detail of the model are provided in “Appendix B.4”). The cell density as a function of the time, shown in Fig. 5, is obtained by integrating the corresponding cell population model according to Eq. (5). The system is initially in a homeostatic condition, meaning that there is a constant influx of cells through differentiation from some upstream stem cell type, which is assumed to be subject to appropriate crowding control, such that this cell influx is constant over time. At a time point $t=0$, all stem cells are removed, which means that the cell influx becomes suddenly zero. Notably, a new homeostatic condition is achieved after a transitory phase thanks to the crowding feedback acting on the C-type. This example demonstrates how an initially committed cell type, i.e. with $\mu _c < 0$, that is regulated via crowding feedback, can become a stem cell type upon removal of the previous stem cell population.

## Discussion

For maintaining healthy adult tissue, the tissue cell population must be maintained in a homeostatic state. Here, we assessed one of the most common generalised regulation mechanisms of homeostasis, which we refer to as *crowding feedback*. Based on this, progenitor cells (stem cells and committed progenitors) adjust their propensities to divide, differentiate, and die, according to the surrounding density of cells of their type, which they sense via biochemical or mechanical signals. For this purpose, we used a generic mathematical model introduced before in reference Parigini and Greulich (2020), Greulich et al. (2021), which describes tissue cell population dynamics in the most generic way, including cell divisions, cell state transitions, and cell loss/differentiation. Based on this model, we rigorously define what is meant when speaking of a ‘homeostatic state’, introducing two notions: *strict* homeostasis is a non-negative steady state of the tissue cell population dynamics, while *dynamical* homeostasis allows, in addition to strict homeostasis, for oscillations and fluctuations, as long as these variations are bounded and a finite long-term average cell population is maintained (such as the endometrium during the menstrual cycle).

By analysing this dynamical system, we find several sufficient and necessary conditions for homeostasis. These conditions are formulated in terms of how the propensities of cell division, differentiation, and cell state changes, of cells whose type is at the apex of an adult cell lineage hierarchy, may depend on their cell density. We find that when, for a wide range of cell density values, the cell division propensity of at least one state decreases with cell density or the differentiation propensity increases with it, while other propensities (e.g. of cell state transitions) are not affected by the cell density, then dynamic homeostasis is assured to prevail (20). For strict homeostasis to be assured, this only needs to be fulfilled at the steady state itself, but in addition, the magnitude of the feedback strength may not be too large (38). We can derive explicit and implicit expressions for this limit on feedback strength for systems of two and three-cell states but cannot do so for arbitrary systems.^9^ Furthermore, we find that a necessary condition for strict homeostasis is that the conditions for dynamic homeostasis are met at least at the steady state cell density.

A direct consequence of the conditions we found is that they allow for a considerable degree of redundancy when more than one propensity depends appropriately on the cell density. Hence feedback pathways, that is, cell dynamics parameters depending on the cell density, may serve as ‘backups’ to each other if one fails. We demonstrate that this confers robustness to the homeostatic system: sometimes one or more crowding feedback pathways may fail, yet the tissue remains in homeostasis.

Finally, we assess how crowding feedback regulation affects the response of committed progenitor cells—which are dividing cells, but are not self-renewing as stem cells are—to a complete depletion of all stem cells. We showed that committed cells which can divide and are under appropriate crowding feedback control (that is, meeting the sufficient conditions (20) and (38) with respect to the density of that cell type), will necessarily, without additional mechanisms or assumptions, reacquire stem cell identity, that is, become self-renewing and being at the apex of the lineage hierarchy. Notably, while this process resembles that of dedifferentiation, it does not involve explicit reprogramming, in that the cell state transitions are reversed. Instead, only the cell fate propensities adjust to the changing environment by balancing proliferation and differentiation as is required for self-renewal. While these are purely theoretical considerations, and such a process has not yet been experimentally found, we predict that it must necessarily occur under the appropriate conditions. This can be measured by assessing the gene expression profiles (e.g. via single-cell RNA sequencing) of cells that ‘dedifferentiate’, i.e. reacquire stemness after depletion of stem cells. Moreover, those considerations yield further, more general insights:

- *Stem cell identity* (also called *stemness*) is neither the property of individual cells nor is it strictly associated with particular cell types, since the same cell type can, through possibly minor adjustments of the pacing of cell division and differentiation, behave as a stem cell or as a committed cell, depending on its environment.
- Instead, we can define *stem cell potential* as the ability of a progenitor cell to respond to its environment via feedback, so that its population is held in a stable steady state (for example by crowding feedback, but other feedback mechanisms may provide this as well). This way, if there are no other cell types higher up in the lineage hierarchy, then it acquires stem cell characteristics, otherwise, it behaves as a committed progenitor cell. Any cell that (1) can divide and differentiate, committed or not, and (2) which responds appropriately to its environment (e.g. by being subject to crowding control) can become a stem cell and thus has ‘stem cell potential’.
- From the latter follows that stemness is a property determined by the environment, not the cell itself.
- ‘Cell plasticity’ might need to be seen in a wider context. Usually, cell plasticity is associated with a change of a cell’s type when subjected to environmental cues, which involves a substantial remodelling of the cell’s morphology and biochemical state. However, we see that a committed cell may turn into a stem cell simply by adjusting the pace of the cell cycle and differentiation processes. This may not require substantial changes in the cell’s morphology or gene expression patterns.

This exemplifies that homeostatic control through crowding feedback is not only a way to render homeostasis stable and robust, but also to create stem cell identities as a collective property of the tissue cell population.

## Figures and Tables

**Fig. 7:**
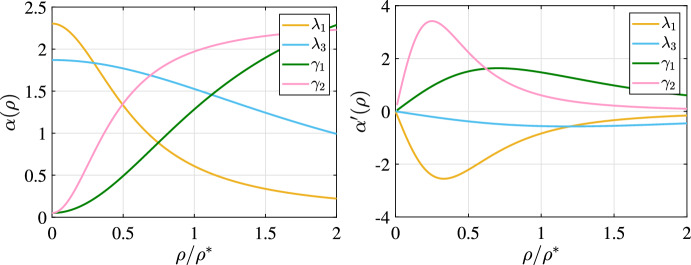
Proliferation and differentiation rates (left panels, with $\alpha$ as a generic placeholder for parameters), and their derivative with respect to $\rho$ (right panels) as functions of cell density normalised by the steady-state $\rho ^*$ for the cell fate model (B15) schematised in Fig. 6. The profiles in the left panel correspond to Hill functions defined in Table 1

**Fig. 8:**
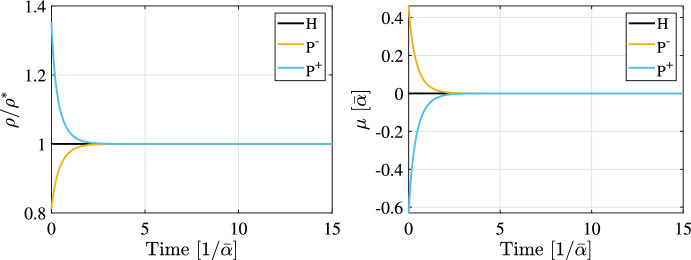
Effect of perturbation of homeostasis under crowding control, when feedback parameters are according to Table 1. (Left) Cell density $\rho$, scaled by the steady-state $\rho ^*$, as a function of time. (Right) Corresponding variation of the dominant eigenvalue $\mu$. Time is scaled by the inverse of $\bar{\alpha } = \min _i{\alpha _i^*}$. Three different initial condition are tested: **H**, corresponds to the steady state $\varvec{\rho }^* = (\rho _1^*,\rho _2^*,\rho _3^*)$, **P**^-^ to $$\begin{pmatrix}0.8 \rho _1^*, 0.75 \rho _2^*, 0.85 \rho _3^* \end{pmatrix}$$ and **P**^+^ to $$\begin{pmatrix}1.5 \rho _1^*, 1.1\rho _1^*, 1.2\rho _1^*\end{pmatrix}$$. Since the steady state is asymptotically stable, thanks to crowding control, the cell population remain in, or return to, a homeostatic state characterised by $\mu = 0$

**Fig. 9:**
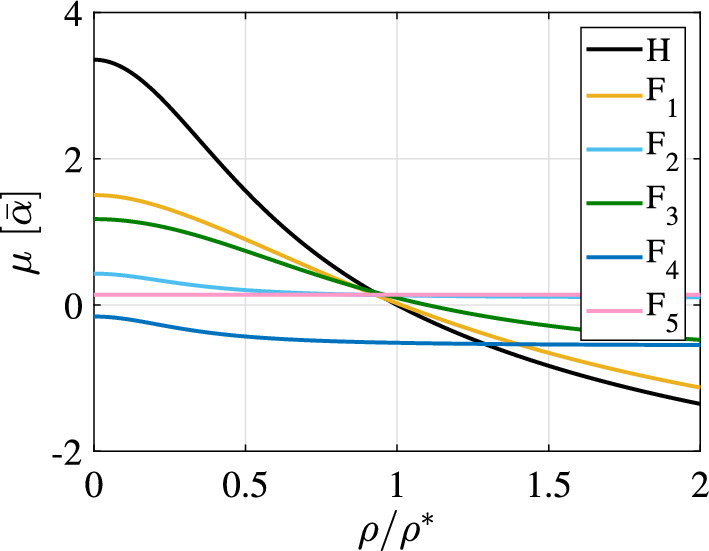
Variation of the dominant eigenvalue $\mu$ as a function of the cell density, $\rho$, normalised by the reference homeostatic state value, $\rho ^*$. The curve **H** corresponds to the reference homeostatic model presented in “Appendix B.1”. The other curves, F_1-5_, represent different sets of feedback failure, as reported in Table 2

**Fig. 10:**
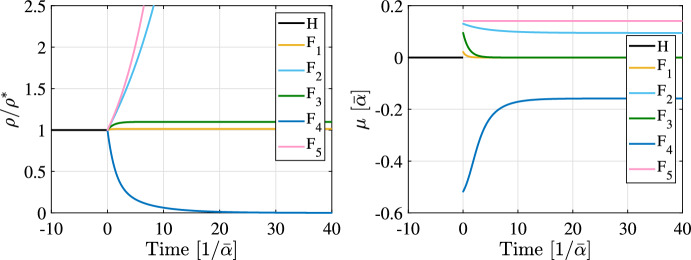
Failure of feedback control for test cases **F**_1,2,3,4_. (Left) Cell density, scaled by the steady state in the homeostatic case, as a function of time. (Right) Corresponding variation of the dominant eigenvalue $\mu$. Time is scaled by the inverse of $\bar{\alpha } = \min _i{\alpha _i^*}$. The homeostatic model, **H**, is perturbed at a time equal to zero to include the feedback failure reported in Table 2. Whilst in **F**_1,3_, the regulation is able to achieve and maintain a new homeostatic state ($\mu = 0$), the remaining cases fail to regulate the cell population, leading to an indefinite growth or shrinking of the tissue

**Fig. 11:**
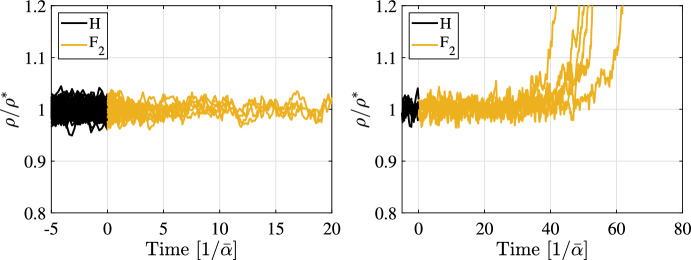
Results of numerical simulations of the stochastic process representing the cell dynamics, according to Sect. B.3. The cell density, scaled by the steady state in the homeostatic case, as a function of the time is shown for 100 random instances. Each shown trajectory is the result of a different instance of the stochastic process. At a time equal to zero, the cell mutation is modelled as a switch of a single random cell from the homeostatic **H** cell dynamics to the **F**_2_ model assessed in “Appendix B.2”. On the left panel, only the trajectories for which the mutated clone goes extinct are shown. The right panel shows the trajectories in which the mutated clone prevails. Dynamics are scaled by $\bar{\alpha }= \min _{i}\{\alpha _i^*\}$

## Appendix A Asymptotic stability assessment based on Routh-Hurwitz

### Background

In control system theory, a commonly used method for assessing the stability of a linear system is the Routh-Hurwitz (RH) criterion (Franklin et al. 2014). It is an algebraic criterion providing a necessary and sufficient condition on the parameters of a dynamic system of arbitrary order to ensure the dynamics are asymptotically stable. In particular, the criterion defines a set of conditions on the coefficients, $p_i$, of the characteristic polynomial, *P*(*s*), written as

A1 $$\begin{aligned} P(s) = s^n + \sum _{i = 1}^n p_i s^{n-i} , \end{aligned}$$

in which *n* corresponds to the dimension of the system. Note that the notation used in this section, based on that from Franklin et al. (2014), is different from that of the main text, where $p_i$ is the polynomial coefficient of *i*th order.

A first result of the RH criterion is that a necessary condition for the dynamical system to be asymptotically stable is that all the coefficients must be positive, that is,

A2 $$\begin{aligned} p_i > 0, \text{ for } \text{ all } i. \end{aligned}$$

Additional conditions on the polynomial coefficients are added for a necessary and sufficient condition. These conditions are based on Routh’s array, written as

A3 $$\begin{aligned} \begin{bmatrix} 1 &{} p_{2} &{} p_{4} &{}\cdots &{} 0\\ p_{1} &{} p_{3} &{}\cdots &{} &{}\\ b_1 &{} b_2 &{}\cdots &{} &{}\\ c_1 &{} &{} &{} &{}\\ \cdots &{} &{} &{} &{} \end{bmatrix}, \end{aligned}$$

in which the first two rows contain all the coefficients of the characteristic polynomial, and the following ones are recursively computed as

A4 $$\begin{aligned} b_i= & {} -\dfrac{\det \begin{pmatrix} 1 &{} p_{2i} \\ p_1 &{} p_{2i+1}\end{pmatrix}}{p_{1}}, \end{aligned}$$

A5 $$\begin{aligned} c_i= & {} -\dfrac{\det \begin{pmatrix} p_1 &{} p_{2i+1} \\ b_1 &{} b_{i}\end{pmatrix}}{b_{1}}, \end{aligned}$$

and so on until a zero is encountered. The RH criterion states that the system is asymptotically stable if and only if the elements in the first column of Routh’s array are positive.

Based on that, it can be easily shown that for a second-order polynomial, the necessary condition (A2) is also sufficient for asymptotic stability (*a.s.*) since $b_1 = p_1 p_2$, which means that

A6 $$\begin{aligned} \text{ The } \text{ system } \text{ is } \text{ a. } \text{ s. } \iff p_i > 0, \text{ for } i = 1, 2 . \end{aligned}$$

Instead, the necessary and sufficient condition for a polynomial of order three results in

A7 $$\begin{aligned} \text{ The } \text{ system } \text{ is } \text{ a. } \text{ s. } \iff p_i> 0, \text{ for } i = 1, 2, 3 \, \text{ and } p_1 p_2 - p_3 > 0 . \end{aligned}$$

The same reasoning can be applied to higher-order dynamics to derive additional conditions on the coefficients $p_i$.

### Verification of the necessary condition for asymptotic stability

The Matlab code for verifying (31) is provided in https://github.com/ cp4u17/Feedback.git.

The strategy used is to evaluate each term in Eq. (31) and simply compare the left and right-hand sides of the equation. We followed a symbolic approach (based on the Matlab symbolic toolbox) for an arbitrary three-state model. A numerical approach was used instead for higher-order dynamics, specifically 4, 5 and 6 state cell fate models. To do so, we randomly defined the cell dynamical matrix at the steady state, $A(\rho ^*)$, and its derivative with respect to $\rho$. Entries were chosen from a uniform distribution and, for assuring a zero dominant eigenvalue for $A(\rho ^*)$, a local optimiser (*fmincon* function of Matlab) was used to find appropriate diagonal elements. For each dimension of the cell fate model, we tested up to 1000 random cases.

### Sufficient condition for asymptotic stability

In this section, we will indicate with the superscripts *A* and *J* the coefficients of the characteristic polynomial expressed as Eq. (A1) respectively of the matrix of the dynamical system, Eq. (6), and those of the Jacobian matrix, Eq. (23).

For a two and three-state system, the following relations can be algebraically derived

A8 $$\begin{aligned} p^J_1 = p^A_1 - \sum _i \eta _i. \end{aligned}$$

where $\eta _i$ is according to Eq. (24). Again, considering that $p^A_1 > 0$, if all $\eta _i \le 0$ then $p^J_1 > 0$.

Hence, the above relation implies that in a two-state system, the RH criterion given by Eq. (A6) is fulfilled when $\varvec{\eta }\le 0$, with at least one negative component (otherwise $J = A$) and therefore the system is asymptotically stable. We recall that asking $\eta _i \le 0$ without further constraints is equivalent to the previously derived condition (38) with $\epsilon _i= \infty$.

For applying the RH criterion to a three-state cell dynamic system, given by Eq. (A7), we need to evaluate the sign of $p^J_2$ and then that of $p^J_1p^J_2 - p^J_3$. To do so, we first write

A9 $$\begin{aligned} p^J_2 = p^A_2 - \sum _i f_i \eta _i, \end{aligned}$$

in which $f_i = \sum _j a_{ji} - Tr(A)$ for $i = 1, 2, 3$. Since the off-diagonal elements are non-negative, and the trace of *A* is negative, then $f_i > 0$ for $i = 1, 2, 3$. This means that if all $\eta _i \le 0$ then $p^J_2 > 0$. Concerning the term $p^J_1p^J_2 - p^J_3$, this can be written as a quadratic form in $$\varvec{\eta }= \begin{pmatrix} \eta _1, \eta _2, \eta _3 \end{pmatrix}$$ as,

A10 $$\begin{aligned} p^J_1p^J_2 - p^J_3 = Q(\varvec{\eta }) = \varvec{\eta }^T A_Q \varvec{\eta }+ \varvec{b}_Q^T \varvec{\eta }+ c_Q, \end{aligned}$$

in which

A11 $$\begin{aligned}{} & {} A_Q = \begin{pmatrix} f_1 &{}\quad f_1 &{}\quad f_1 \\ f_2 &{}\quad f_2 &{}\quad f_2 \\ f_3 &{}\quad f_3 &{}\quad f_3\end{pmatrix} \,, \end{aligned}$$

A12 $$\begin{aligned}{} & {} \varvec{b}_Q = -p^A_1 \begin{pmatrix} f_1 \\ f_2 \\ f_3 \end{pmatrix} - \dfrac{p_{2}^A}{\varvec{v} \varvec{w}}\begin{pmatrix} v_3 (w_3 - w_1) + v_2 (w_2 - w_1) \\ v_3 (w_3 - w_2) + v_1 (w_1 - w_2) \\ v_2 (w_2 - w_3) + v_1 (w_1 - w_3) \end{pmatrix} \,, \end{aligned}$$

and $c_Q = p_1^Ap_2^A$. Here, $\varvec{v}=(v_1,v_2,v_3)$ is a left dominant eigenvector and $\varvec{w}=(w_1,w_2,w_3)$ a right dominant eigenvector.

We now note that the matrix $A_Q$ is semidefinite positive since two eigenvalues are zero (the rows are two-fold degenerate) and one is positive, equal to $Tr(A_Q) = \sum _i f_i$, and $c_Q>0$. We now distinguish two cases, depending on the sign of $\varvec{b}_Q$ elements. First, if $\varvec{b}_Q \le 0$, then $Q(\varvec{\eta }) > 0$ for any $\varvec{\eta }\le 0$. Since $f_i,p_1^A,p_2^A, \varvec{v} \varvec{w} > 0$, we get a sufficient condition for $\varvec{b}_Q \le 0$, namely,

A13 $$\begin{aligned} 0&\le v_3 (w_3 - w_1) + v_2 (w_2 - w_1) \nonumber \\ 0&\le v_3 (w_3 - w_2) + v_1 (w_1 - w_2) \nonumber \\ 0&\le v_2 (w_2 - w_3) + v_1 (w_1 - w_3). \end{aligned}$$

In that case, asymptotic stability and thus crowding feedback control is assured for any $\varvec{\eta }<0$, and thus the bound for feedback strength is $\epsilon _i = \infty$ for $i=1,2,3$.

Otherwise, if there is at least one positive element in $\varvec{b}_Q$, then $Q(\varvec{\eta }) > 0$ only if $|\eta _i |< \epsilon _i$, where $\varvec{\epsilon }= (\epsilon _1,\epsilon _2,\epsilon _3)$ are the absolute values of the solutions to the equation $Q(\varvec{\eta }) = 0$, that is—given that $\eta _i$ are negative—the solution to,

A14 $$\begin{aligned} 0 = \varvec{\epsilon }^T A_Q \varvec{\epsilon }- \varvec{b}_Q^T \varvec{\epsilon }+ c_Q\,. \end{aligned}$$

Importantly, we note that the elements of $\varvec{b}_Q$ depend uniquely on the properties of the dynamical system and therefore, they can be determined without requiring the knowledge of the parameter derivatives, i.e. the specific crowding feedback dependencies.

The Matlab code for verifying (A8), (A9) and (A10) is provided in https://github.com/cp4u17/Feedback.git.

## Appendix B Test case

### Asymptotic stability

This section reports the details of the model used for numerical examples presented in the main text. The cell dynamics correspond to the following three-state cell fate model

B15 $$\begin{aligned} \begin{aligned}&X_{1} \xrightarrow {\lambda _{1}} X_{1}+X_{1}, \qquad X_{1} \xrightarrow {\omega _{13}} X_{3}, \qquad X_{1} \xrightarrow {\gamma _{1}} \emptyset \\&X_{2} \xrightarrow {\omega _{21}} X_{1}, \qquad X_{2} \xrightarrow {\omega _{23}} X_{3}, \qquad X_{2} \xrightarrow {\gamma _{2}} \emptyset \\&X_{3} \xrightarrow {\lambda _{3}} X_{3}+X_{3}, \qquad X_{3} \xrightarrow {\omega _{31}} X_{1}, \qquad X_{3} \xrightarrow {\omega _{32}} X_{2}, \end{aligned} \end{aligned}$$

whose network is shown in Fig. 6. In such a model, for simplicity, we only consider symmetric self-renewing divisions so that $\kappa _{ij} = \omega _{ij}$. Also, we apply the crowding feedback to division rates, $\lambda _i$, and differentiation rates $\gamma _i$. In this way, it is straightforward to apply the sufficient condition (38) for asymptotic stability since $\kappa _{ij}' = 0$ for all *i*, *j*.

Hence, each kinetic parameter of the type $\alpha _i \in \{\lambda _j,\gamma _j\}_{j=1,\ldots ,3}$ is expressed as a function of $\rho$, whilst those of the type $\alpha _i \in \{\kappa _{jk}\}_{j,k=1,\ldots ,3}$ are constant. In particular, we chose a Hill function (Lei et al. 2014) where $\alpha _i(\rho ) = c_i + k_i \rho ^{n_i}/(K_i^{n_i} + \rho ^{n_i})$ in case $\alpha _i$ is a differentiation rate, so that $\alpha _i' = \partial \alpha _i / \partial \rho > 0$, and $\alpha _i(\rho ) = c_i + k_i/ (K_i^{n_i} + \rho ^{n_i})$ in case it is a proliferation rate, so that $\alpha _i' < 0$. According to (38) this choice assures that, if there is a value $\rho = \rho ^*$ for which $\mu (\rho ^*) = 0$, this corresponds to an asymptotically stable steady state.

The parameter values used in our example are reported in Table 1, and the profiles of the proliferation and differentiation rates as a function of $\rho$ are shown in Fig. 7. Based on these values, the steady state corresponds to $\rho ^* = 1$ (arbitrary unit). As expected, the dominant eigenvalue of the Jacobian at the steady state is negative ($\mu _J = -1.21$).

To test the dynamical behaviour of the tissue cell population, we numerically solved the system of ODEs (5) for different initial conditions based on the explicit Runge–Kutta Dormand–Prince method (Matlab *ode45* function). The results are shown in Fig. 8 as the time evolution of $\rho$, normalised by the steady-state $\rho ^*$, (left panels), and of the dominant eigenvalue, $\mu$ (right panels). The label **H** indicates an initial condition corresponding to the self-renewing state $\varvec{\rho }^*$, that is, the system is initially in homeostasis. In the simulations labelled as **P**^-^ and **P**^+^, we applied perturbation in the initial state $\varvec{\rho }^* = (\rho _1^*,\rho _2^*,\rho _3^*)$, which are, respectively, $$\begin{pmatrix}0.8 \rho _1^*, 0.75 \rho _2^*, 0.85 \rho _3^* \end{pmatrix}$$ and $$\begin{pmatrix}1.5 \rho _1^*&1.1 \rho _2^*&1.2 \rho _3^* \end{pmatrix}$$. As expected, in all these cases, the feedback’s effect is stabilising the system so that it returns to the steady state upon perturbation, $\varvec{\rho }\rightarrow \varvec{\rho }^*$, (asymptotic stability) and thus regains self-renewal property, $\mu \rightarrow 0$, over time.

**Table 1:** Values of the Hill function parameters describing the kinetic parameters in case of homeostasis regulation via crowding feedback for the cell fate model (B15). The generic kinetic parameters (represented as $\alpha _i$ in the right columns of the table) are a function of the total cell density, $\rho$, and are given by $\gamma _i(\rho ) = c + k \rho ^{n}/(K^{n} + \rho ^{n})$ and $\lambda _i(\rho ) = c + k/(K^{n} + \rho ^{n})$ with $i=1,2,3$. A common value $c = 0.05$ is assumed. State transition rates $\omega _{ij}$, are constant and equal to $\kappa _{ij}$. For such a cell fate dynamics, the steady state is $\rho ^* = 1$. The unit of the kinetic parameter is arbitrary and therefore omitted. Unless specified otherwise, these values apply to all the numerical examples presented in this work

	*k*	*K*	*n*	$\alpha ^*$	$\alpha '$
$\lambda _1$	0.74	0.57	2.00	0.61	$-$0.84
$\lambda _3$	7.79	2.07	2.00	1.53	$-$0.56
$\gamma _1$	3.07	1.22	2.00	1.28	1.48
$\gamma _2$	2.28	0.43	2.00	1.97	0.61
$\kappa _{13}$	–			0.95	0.00
$\kappa _{21}$	–			1.44	0.00
$\kappa _{23}$	–			1.71	0.00
$\kappa _{31}$	–			2.03	0.00
$\kappa _{32}$	–			1.35	0.00

### Failure of feedback function

Based on the cell fate model regulated via crowding feedback described in the previous section, we assess the impact of failure in one or more feedback functions. In particular, the failure of the crowding regulation is modelled, assuming one or more kinetic parameters as a constant. Five different failure test cases are assessed. For doing so, we chose $\alpha _i = (1+C) \alpha _i^*$ being constant instead of depending on $\rho$, in which $\alpha ^*$ is the value at the steady state when there are no failures (reported in Table 1) and *C* is a constant (reported in Table 2). Five test cases, indicated as **F**_1-5_, are assessed.

In test case **F**_1_, only one feedback fails. Three of the four kinetic parameters fail in cases **F**_2-4_. Finally, **F**_5_ represents a case where all the feedback functions fail. The corresponding variability of the dominant eigenvalue, $\mu$, as a function of the cell density is shown in Fig. 9. It is clear that whilst **F**_1-4_ cases satisfy the sufficient condition for strict homeostasis, (38), in test case **F**_5_, the dominant eigenvalue being constant means that there is no homeostatic regulation. Importantly, there is no steady state in test cases **F**_2,4_ since the dominant eigenvalue is always positive in one case or negative in the other.

**Table 2:** Value of the constant *C* in the feedback failure test cases. Whenever a failure in the feedback of one kinetic parameter $\alpha$ occurs, that parameter is modelled as a constant, $\alpha = (1+C)\alpha ^*$, for which the steady-state value, $\alpha ^*$, is reported in Table 1. Test cases F_1_ and F_2_ correspond to those presented in the main text (Fig. 2)

Parameter	**F**_1_	**F**_2_	**F**_3_	**F**_4_	**F**_5_
$\lambda _1$	$+5\%$	$+5\%$	$+5\%$	$-20\%$	$-5\%$
$\lambda _3$	–	$+5\%$	$+5\%$	$-20\%$	$-5\%$
$\gamma _1$	–	$-5\%$	–	$+20\%$	$-5\%$
$\gamma _2$	–	–	$-5\%$	–	$-5\%$

Based on these assumptions, we numerically solved the system of ODEs (5) using the explicit Runge–Kutta Dormand–Prince method (Matlab *ode45* function). The failure test cases start at time $t=0$ from an initially homeostatic condition, **H**. The results are shown in Fig. 10 as the time evolution of $\rho$, normalised by the homeostatic steady-state $\rho ^*$, (left panels), and of the dominant eigenvalue, $\mu$ (right panels). Note that the cases **F**_1,2_ correspond respectively to the **Single failure** and **Multiple failures** reported in the main text (Fig. 2).

In two cases, **F**_1,3_, despite a single or multiple feedback functions failing, a new homeostatic condition is reached after some time, where then $\mu = 0$. However, suppose a different set of feedback fails, like in **F**_2,4_, such that the dominant eigenvalue is respectively positive or negative for all $\rho$. In that case, no steady state can be attained, and the tissue cell population will hyper-proliferate or decline in the long term. Hence, even if the condition for asymptotic stability is met, there is no steady state. Finally, if homeostasis is not regulated at all, as in **F**_5_, then the population dynamics only depend on the value of the dominant eigenvalue (the cell dynamical model (5) turns linear). In the case shown, $\mu > 0$ and therefore, the cell population diverges.

### Single cell mutation scenario

To assess the tissue dynamics with a single-cell mutation, as presented in the main text, we modelled the clonal dynamics, namely, the dynamics of single cells and their progeny. For doing so, we considered the model (B15) as a Markov process with the same numerical rates as before, but now events are treated as stochastic. Then, we run numerical simulations using the Gillespie algorithm (Gillespie 1977) to evaluate this model. In particular, the results presented in this work are based on 100 independent instances, where each instance is a possible realisation of the stochastic process. We chose a total cell number $N_0 = 5000$ as the initial condition (cell density is based on unitary volume). In real tissues, the number of cells could be a few orders of magnitude larger. However, this number is sufficiently large to avoid the extinction of the process in the time scale analysed, so once rescaled, these dynamics are representative of those in the tissue. All the simulations are stopped when the mutated clone goes extinct or divergence of the dynamics is detected, defined as reaching $N=5 N_0$.

From an implementation point of view, we consider a cell fate model represented by two disconnected cell state networks to model the tissue dynamics, including the mutated cell. One network corresponds to the unperturbed test case **H**, and the other to the dysregulated one, **F**_2_ (both described in “Appendix B.2”). The simulation starts with $N_0$ cells in the **H** network, distributed in each state proportionally to the expected steady-state distribution in the tissue, and no cells in the **F**_2_ network. Thus, since the two networks are disconnected, **F**_2_ remains empty, and the simulation represents the tissue dynamics before the dysregulation. At a time equal to zero, we moved one cell from a random state in the **H** network to the corresponding state in the **F**_2_ one. This simulation represents the tissue dynamics, including the single mutated cell.

In Fig. 11 (left), all the trajectories where the mutated clones go extinct are shown. In these cases, the tissue dynamics remain globally unaffected by the mutation. Due to the stochastic nature of the process, mutant clones can go extinct even if the growth parameter is positive. That is, even in cases where divergence would be observed for the tissue-wide disruption. However, this does not occur in all the instances. The right panel of the same figure shows those instances where the mutated clone does not go extinct and eventually prevails, resulting in diverging cell population dynamics. For the chosen parameters, this divergence of the mutated clone is detected in 6$\%$ of all cases. Surprisingly, only a few clones survive despite a proliferative advantage, but this is plausible for a small fitness advantage (For example, in the case of a single state with cell division rate $\lambda$ and loss rate $\gamma$—a simple branching process (Haccou et al. 2005)—the probability for the a mutant with $\mu > 0$, that is, $\lambda > \gamma$, to establish is $1 - \gamma /\lambda$, which can be very low for $\lambda \approx \gamma$).

In the main text (Fig. 3), only one profile for each scenario is shown, respectively. They correspond to instance $\#24$ for the homeostatic case and instance $\#43$ for the diverging case.

### Quasi-dedifferentiation

The numerical example presented in the main text is based on the same cell fate model described in “Appendix B.1”. We assume that the generic kinetic parameter, $\alpha _i$, is a function of the total cell density. This is $\alpha _i(\rho ) = \alpha _i(\rho _s + \rho _c)$, where $\rho _s$ and $\rho _c$ are the cell density respectively of the stem and committed cell types. This model represents a typical situation where the competition of growth signalling factors dries the proliferative and differentiation rates.

To model the dynamics of a committed cell type, we choose a constant non-negative $$\varvec{u} = \begin{pmatrix}0.02&0.07&0.06\end{pmatrix}^T$$ to model for the cell influx. We also consider that the global differentiation rate of stem cells into committed cells is unitary so that $\varvec{\rho }_s^* = \varvec{u}$. For such a model, the steady state, $\rho _c^*$, is asymptotically stable.

The figures presented in the main text are based on the numerical integration of the system of ordinary differential equation (40). In particular, we used the explicit Runge–Kutta Dormand–Prince method (Matlab *ode45* function).

## Footnotes

1 More precisely, $$\alpha _i\vert _{i=1,..,m}:= \lambda _i$$, $$\alpha _i\vert _{i=m+1,..,2\,m}:= \gamma _{i-m}$$, $$\alpha _i\vert _{i=2\,m+1,..,2\,m+m^2}:= \kappa _{\lfloor (i-2\,m)/m \rfloor ,i-\lfloor (i-2\,m)/m \rfloor m}$$

2 In Greulich et al. (2021), this condition, defined through dependency on cell number, can be directly translated into a condition on the cell density derivative if the volume is assumed as a constant.

3 Note that $a_{ii}$ is always negative or zero

4 The diagonal elements of the random matrix are tuned using a local optimiser (*fmincon* function of Matab) so that the matrix has a zero dominant eigenvalue.

5 Only in this example, feedback control fails upon multiple failures; in general, multiple failures could still be compensated to maintain homeostatic control.

6 For example, in the case of a single state with cell division rate $\lambda$ and loss rate $\gamma$—a simple branching process—the probability for a mutant with $\mu > 0$, that is, $\lambda > \gamma$, to establish is $1-\gamma /\lambda$, which is less than certain.

7 While a Markov process is an approximation which not necessarily reflects the probability distribution of subsequent event times realistically, it is often sufficient to assess the qualitative behaviour of a system with low numbers, subject to random influences from the environment.

8 This can be seen when multiplying the steady state condition for (40), $A_c^*(\rho _s,\rho _c) \varvec{\rho }_c + \varvec{u}^* = 0$ with a positive left dominant eigenvector $\varvec{v}$, giving, $\mu _c \varvec{v} \varvec{\rho }^*_c + \varvec{v} \varvec{u}^* = 0$. Since $\varvec{\rho }^*$ and $\varvec{v}$ have all positive entries and $\varvec{u}^*$ is non-negative, this equation can only be fulfilled for $\mu _c<0$.

9 Note that since this is a sufficient and not a necessary condtion, homeostasis may prevail even if feedback is stronger than this limit, but it cannot be sufficiently assured through the conditions we found. Other, less restrictive conditions, could possibly be found in the future.

## References

[CR1] Alarcon T, Marciniak-Czochra A (2011) A model for stem cell population dynamics with regulated maturation delay. Discrete Contin Dyn Syst 32–43

[CR2] Bin Z, Karen T, Kun-Liang G (2011) The Hippo pathway in organ size control, tissue regeneration and stem cell self-renewal. Nature Cell Biol 13(8):877–883. arXiv:NIHMS150003. 10.1038/ncb2303.The 10.1038/ncb2303 PMC3987945 21808241

[CR3] Bocharov G, Quiel J, Luzyanina T, Alon H, Chiglintsev E, Chereshnev V, Meier-Schellersheim M, Paul WE, Grossman Z (2011). Feedback regulation of proliferation vs. differentiation rates explains the dependence of CD4 T-cell expansion on precursor number. Proc Natl Acad Sci USA.

[CR4] Chatzeli L, Simons BD (2020). Tracing the dynamics of stem cell fate. Cold Spring Harbor Perspect Biol.

[CR5] Colom B, Jones PH (2016). Clonal analysis of stem cells in differentiation and disease. Curr Opin Cell Biol.

[CR6] Donati G, Rognoni E, Hiratsuka T, Liakath-Ali K, Hoste E, Kar G, Kayikci M, Russell R, Kretzschmar K, Mulder KW, Teichmann SA, Watt FM (2017). Wounding induces dedifferentiation of epidermal Gata6+ cells and acquisition of stem cell properties. Nat Cell Biol.

[CR7] Eisenhoffer GT, Rosenblatt J (2013). Bringing balance by force: Live cell extrusion controls epithelial cell numbers. Trends Cell Biol.

[CR8] Eisenhoffer GT, Loftus PD, Yoshigi M, Otsuna H, Chien C-B, Morcos PA, Rosenblatt J (2012). Crowding induces live cell extrusion to maintain homeostatic cell numbers in epithelia. Nature.

[CR9] Franklin GF, Powell JD, Emami-Naeini A (2014). Feedback control of dynamic systems.

[CR10] Gillespie DT (1977). Exact stochastic simulation of coupled chemical reactions. J Phys Chem.

[CR11] Greulich P, Simons BD (2016). Dynamic heterogeneity as a strategy of stem cell self-renewal. Proc Natl Acad Sci.

[CR12] Greulich P, MacArthur BD, Parigini C, Sánchez-García RJ (2019). Stability and steady state of complex cooperative systems: a diakoptic approach. R Soc Open Sci.

[CR13] Greulich P, MacArthur BD, Parigini C, Sánchez-García RJ (2021). Universal principles of lineage architecture and stem cell identity in renewing tissues. Development.

[CR14] Gudipaty SA, Lindblom J, Loftus PD, Redd MJ, Edes K, Davey CF, Krishnegowda V, Rosenblatt J (2017). Mechanical stretch triggers rapid epithelial cell division through Piezo1. Nature.

[CR15] Haccou P, Jagers P, Vatutin VA (2005). Branching processes: variation, growth, and extinction of populations.

[CR16] Hara K, Nakagawa T, Enomoto H, Suzuki M, Yamamoto M, Simons BD, Yoshida S (2014). Mouse spermatogenic stem cells continually interconvert between equipotent singly isolated and syncytial states. Cell Stem Cell.

[CR17] Horn RA, Johnson CR (1985). Matrix analysis.

[CR18] Hufnagel L, Teleman AA, Rouault H, Cohen SM, Shraiman BI (2007). On the mechanism of wing size determination in fly development. Proc Natl Acad Sci.

[CR19] Johnston MD, Edwards CM, Bodmer WF, Maini PK, Chapman SJ (2007). Mathematical modeling of cell population dynamics in the colonic crypt and in colorectal cancer. Proc Natl Acad Sci.

[CR20] Jopling C, Boue S, Belmonte JCI (2011). Dedifferentiation, transdifferentiation and reprogramming: three routes to regeneration. Nat Rev Mol Cell Biol.

[CR21] Jörg DJ, Kitadate Y, Yoshida S, Simons BD (2019) Competition for stem cell fate determinants as a mechanism for tissue homeostasis. arXiv arXiv:1901.03903

[CR22] Kitadate Y, Jörg DJ, Tokue M, Maruyama A, Ichikawa R, Tsuchiya S, Segi-Nishida E, Nakagawa T, Uchida A, Kimura-Yoshida C, Mizuno S, Sugiyama F, Azami T, Ema M, Noda C, Kobayashi S, Matsuo I, Kanai Y, Nagasawa T, Sugimoto Y, Takahashi S, Simons BD, Yoshida S (2019). Competition for mitogens regulates spermatogenic stem cell homeostasis in an open niche. Cell Stem Cell.

[CR23] Lander AD, Gokoffski KK, Wan FYM, Nie Q, Calof AL (2009). Cell lineages and the logic of proliferative control. PLoS Biol.

[CR24] Lei J, Levin SA, Nie Q (2014). Mathematical model of adult stem cell regeneration with cross-talk between genetic and epigenetic regulation. Proceedings of the National Academy of Sciences.

[CR25] MacCluer CR (2000). The many proofs and applications of Perron’s theorem. SIAM Rev.

[CR26] Marinari E, Mehonic A, Curran S, Gale J, Duke T, Baum B (2012). Live-cell delamination counterbalances epithelial growth to limit tissue overcrowding. Nature.

[CR27] McClatchey AI, Yap AS (2012). Contact inhibition (of proliferation) redux. Curr Opin Cell Biol.

[CR28] Merrell AJ, Stanger BZ (2016). Adult cell plasticity in vivo: De-differentiation and transdifferentiation are back in style. Nat Rev Mol Cell Biol.

[CR29] Murata K, Jadhav U, Madha S, van Es J, Dean J, Cavazza A, Wucherpfennig K, Michor F, Clevers H, Shivdasani RA (2020). Ascl2-dependent cell dedifferentiation drives regeneration of ablated intestinal stem cells. Cell Stem Cell.

[CR30] Nakagawa T, Jörg DJ, Watanabe H, Mizuno S, Han S, Ikeda T, Omatsu Y, Nishimura K, Fujita M, Takahashi S, Kondoh G, Simons BD, Yoshida S, Nagasawa T (2021). A multistate stem cell dynamics maintains homeostasis in mouse spermatogenesis. Cell Rep.

[CR31] National Institute of Health: Stem Cell Basics (2016). https://stemcells.nih.gov/info/basics

[CR32] Nonomura K, Hirata H (2020). Cell mechanosensing underlies homeostasis of multicellular systems. Biophys Physicobiol.

[CR33] Parigini C (2022) Mathematical modelling of cell fate dynamics in homeostasis. Ph.D. thesis, University of Southampton

[CR34] Parigini C, Greulich P (2020) Universality of clonal dynamics poses fundamental limits to identify stem cell self-renewal strategies. eLife 9:1–44. 10.7554/eLife.56532 10.7554/eLife.56532 PMC7444910 32687057

[CR35] Puliafito A, Hufnagel L, Neveu P, Streichan S, Sigal A, Fygenson DK, Shraiman BI (2012). Collective and single cell behavior in epithelial contact inhibition. Proc Natl Acad Sci.

[CR36] Puri S, Folias AE, Hebrok M (2015). Plasticity and dedifferentiation within the pancreas: development, homeostasis, and disease. Cell Stem Cell.

[CR37] Ritsma L, Ellenbroek SIJJ, Zomer A, Snippert HJ, De Sauvage FJ, Simons BD, Clevers H, Van Rheenen J (2014). Intestinal crypt homeostasis revealed at single stem cell level by in vivo live-imaging. Nature.

[CR38] Rodilla V, Fre S (2018). Cellular plasticity of mammary epithelial cells underlies heterogeneity of breast cancer. Biomedicines.

[CR39] Scadden DT (2006). The stem-cell niche as an entity of action. Nature.

[CR40] Shraiman BI (2005). Mechanical feedback as a possible regulator of tissue growth. Proc Natl Acad Sci.

[CR41] Stiehl T, Marciniak-Czochra A (2011). Characterization of stem cells using mathematical models of multistage cell lineages. Math Comput Model.

[CR42] Stiehl T, Marciniak-Czochra A (2017). Stem cell self-renewal in regeneration and cancer: insights from mathematical modeling. Curr Opin Syst Biol.

[CR43] Tata PR, Rajagopal J (2016). Cellular plasticity: 1712 to the present day. Curr Opin Cell Biol.

[CR44] Tata PR, Mou H, Pardo-Saganta A, Zhao R, Prabhu M, Law BM, Vinarsky V, Cho JL, Breton S, Sahay A, Medoff BD, Rajagopal J (2013). Dedifferentiation of committed epithelial cells into stem cells in vivo. Nature.

[CR45] Tetteh PW, Farin HF, Clevers H (2015). Plasticity within stem cell hierarchies in mammalian epithelia. Trends Cell Biol.

[CR46] Tetteh PW, Basak O, Farin HF, Wiebrands K, Kretzschmar K, Begthel H, van den Born M, Korving J, De Sauvage FJ, van Es JH, Van Oudenaarden A, Clevers H (2016). Replacement of lost Lgr5-positive stem cells through plasticity of their enterocyte-lineage daughters. Cell Stem Cell.

[CR47] Tomasetti C, Vogelstein B, Parmigiani G (2013). Half or more of the somatic mutations in cancers of self-renewing tissues originate prior to tumor initiation. Proc Natl Acad Sci.

[CR48] Varga RS (2000). Matrix iterative analysis.

[CR49] Watt FM, Hogan LM (2000). Out of Eden: stem cells and their niches. Science.

[CR50] Yu FX, Mo JS, Guan KL (2012). Upstream regulators of the Hippo pathway. Cell Cycle.

